# A selective autophagy receptor VISP1 induces symptom recovery by targeting viral silencing suppressors

**DOI:** 10.1038/s41467-023-39426-0

**Published:** 2023-06-29

**Authors:** Xin Tong, Jia-Jia Zhao, Ya-Lan Feng, Jing-Ze Zou, Jian Ye, Junfeng Liu, Chenggui Han, Dawei Li, Xian-Bing Wang

**Affiliations:** 1 State Key Laboratory of Plant Environmental Resilience, College of Biological Sciences, China Agricultural University, 100193 Beijing, China; 2 College of Plant Protection, China Agricultural University, 100193 Beijing, China; 3 State Key laboratory of Plant Genomics, Institute of Microbiology, Chinese Academy of Sciences, Beijing, China

**Keywords:** Microbe, Virus-host interactions, Plant cell biology

## Abstract

Selective autophagy is a double-edged sword in antiviral immunity and regulated by various autophagy receptors. However, it remains unclear how to balance the opposite roles by one autophagy receptor. We previously identified a virus-induced small peptide called VISP1 as a selective autophagy receptor that facilitates virus infections by targeting components of antiviral RNA silencing. However, we show here that VISP1 can also inhibit virus infections by mediating autophagic degradation of viral suppressors of RNA silencing (VSRs). VISP1 targets the cucumber mosaic virus (CMV) 2b protein for degradation and attenuates its suppression activity on RNA silencing. Knockout and overexpression of *VISP1* exhibit compromised and enhanced resistance against late infection of CMV, respectively. Consequently, VISP1 induces symptom recovery from CMV infection by triggering 2b turnover. VISP1 also targets the C2/AC2 VSRs of two geminiviruses and enhances antiviral immunity. Together, VISP1 induces symptom recovery from severe infections of plant viruses through controlling VSR accumulation.

## Abstract (web summary)

Symptom recovery is induced by a balanced “arms race” between viruses and plants. Here, the authors show that a small peptide mediates autophagic degradation of viral silencing suppressors to reach the balance of virus pathogenicity and plant immunity.

## Introduction

Autophagy is a conserved quality control process through degrading damaged and unwanted components during cell differentiation, development, starvation, biotic and abiotic stresses^1,2^. In nonselective autophagy, bulk cellular portions are recruited into the phagophore for degradation, recycling nutrient into the cytoplasm during starvation^3^. However, accumulating evidence indicates that most of substrates are degraded in a selective manner^3^. Selective autophagy receptors recognize specific substrates and interact with the ATG8 family proteins in the phagophore, and result in substrate degradation^4^. Most receptors contain an ATG8-interacting motif (AIM) to interact with the core autophagy proteins ATG8s in the phagophore^5^. Besides, a class of receptors have recently been identified to interact with ATG8s through a ubiquitin-interacting motif (UIM)^6–8^. Identification of further selective receptors and their specific cargoes will improve our understanding of selective autophagy functions.

Over the past decades, numerous studies have demonstrated double-edged sword roles of selective autophagy in plant virus infections^9–11^. Selective autophagy functions as a key regulator of innate antiviral immunity by degrading viral components and/or host factors essential for virus infections. Selective autophagy receptors are master players in these processes through specifically targeting various cargoes. For instance, the neighbor of BRCA 1 (NBR1), a conserved cargo receptor in animal and plants, directly targets the viral capsid protein of cauliflower mosaic virus (CaMV) and the helper component protease HC-Pro protein of turnip mosaic virus (TuMV)^12,13^. Besides, ATG6/Beclin1 directly interacts with the GDD motif of the TuMV polymerase protein for autophagic degradation^14^. A protein interacting with the P3 of rice stripe virus (RSV), P3IP, was identified as a new cargo receptor to mediate autophagic degradation of the RSV P3 protein in *Nicotiana benthamiana*^15^. In addition, the cotton leaf curl Multan virus βC1 protein and the tomato leaf curl Yunnan virus nucleoprotein C1 protein are degraded through direct interaction with ATG8s^16,17^.

To survive from the ongoing “arms race” with host plants, plant viruses usually manipulate autophagy to degrade antiviral agents, thereby inhibiting antiviral defenses and facilitating virus infections. ARGONAUTE1 (AGO1) and RNA-dependent RNA polymerase 6 (RDR6)/suppressor of gene silencing 3 (SGS3) bodies, two core components of antiviral RNA silencing, are targeted for autophagic degradation, which is manipulated by plant viruses^18–22^. In addition, the multifunctional γb protein of barley stripe mosaic virus (BSMV) inhibits antiviral autophagy by interfering with the interaction of ATG7–ATG8^23^. Recently, the BSMV γa protein has been found to inhibit vacuolar acidification and autophagy degradation^24^. Although significant progress has been made, it remains largely unknown how plant viruses fine-tune double-edged roles of autophagy to achieve virus long-term infections in plants.

RNA silencing-mediated antiviral immunity is evolutionarily conserved in eukaryotes. Viral double-stranded RNA is processed by host Dicers, and the resultant siRNAs guide specific cleavage of invading viral RNAs through binding AGO proteins^25,26^. In plants, RNAi-based antiviral immunity requires viral siRNA amplification by host RDR1 and RDR6^27^. Besides, SGS3 and its partner RDR6 form SGS3/RDR6 bodies as siRNA amplification centers^28,29^. As a counter defense strategy, most of plant RNA and DNA viruses encode viral suppressors of RNA silencing (VSRs) to counteract RNA silencing^30,31^. CMV 2b is a well-known VSR of post transcriptional gene silencing and transcriptional gene silencing through dsRNA binding, interacting with AGOs, and suppression of RDR-dependent siRNA amplification^32–35^. Both CMV 2b and SGS3/RDR6 bodies are targeted by selective autophagy, and 2b in turn inhibit selective autophagy^7,36,37^. However, how to finely balance this cross-talk process remains to be determined.

A great number of small open reading frames (ORFs, 30-100 amino acids) hidden in plant genomes have been ignored for a long time but are recently characterized in plant development and immunity^38,39^. Recently, we identified a CMV-induced sORF composing of 71 amino acids, termed VIRUS-INDUCED SMALL PEPTIDE 1 (VISP1). VISP1 contains an ATG8-interacting UIM domain and acts as a selective autophagy receptor^7^. VSR-deficient virus mutants have been efficiently used to define particular RNAi genes in previous studies^27,40–43^. For instance, an alanine-substitution mutant of CMV 2b in its N-terminal 15th leucine and 18th methionine (2blm) is severely compromised in its VSR activity and cannot suppress SGS3/RDR6-mediated RNAi amplification^41^. Therefore, CMV-2blm is a VSR-deficient virus mutant and exhibits very weak pathogenicity^41^. We previously used CMV-2blm to reveal the pro-viral function of VISP1 via degradation of SGS3/RDR6 bodies^7^.

Here, we further explored whether the selective receptor VISP1 targeted viral proteins other than host SGS3/RDR6 bodies. We found that VISP1 targeted some VSRs, like the 2b protein of CMV, the P14 protein of pothos latent virus (PoLV), the C2 protein of beet severe curly top virus (BSCTV) and the AC2 protein of cabbage leaf curl virus (CaLCuV). VISP1 exhibited antiviral activity against these viruses, especially along with increasing accumulation of VSRs in late infections. Therefore, we propose that VISP1 plays double-edged sword roles to balance plant resistance and virus pathogenicity in the ongoing “arms race” between host plants and viruses.

## Results

### VISP1 interacts with CMV 2b and drives it into autophagosomes

**Fig. 1:**
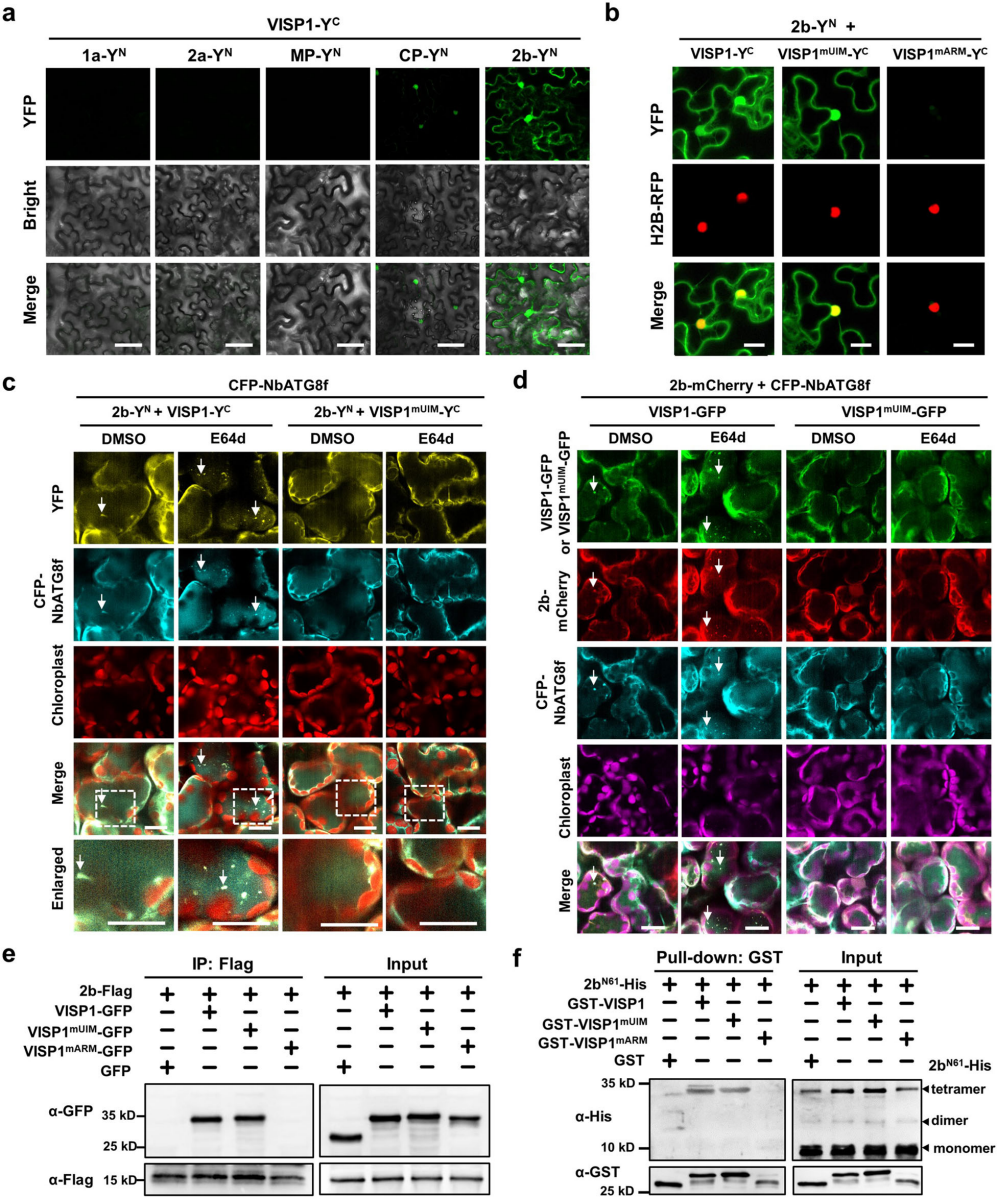
VISP1 interacts with CMV 2b and drives it into autophagosomes. **a** Bimolecular fluorescence complementation (BiFC) analyses of interactions between VISP1 and CMV 1a/2a/MP/CP/2b proteins. *N. benthamiana* leaves were photographed at 60 h post-infiltration (hpi) with agroinfiltration for expression of indicated proteins fused with the N (Y^N^) and C (Y^C^) terminal halves of YFP, respectively. Scale bars, 50 μm. **b** BiFC analyses of interactions between CMV 2b and VISP1/VISP1^mUIM^ /VISP1^mARM^ in H2B-RFP-transgenic *N. benthamiana* leaves at 60 hpi. H2B-RFP is a nuclear marker. Scale bars, 20 μm. **c** Confocal analyzing co-localization of the 2b-Y^N^/VISP1-Y^C^-formed bodies with CFP-NbATG8f-labelled autophagic bodies in *N. benthamiana* leaves. VISP1^mUIM^-Y^C^ served as a negative control. The infiltrated leaves were treated with 100 µM E64d or DMSO at 48 hpi and photographed at 60 hpi. Scale bars, 20 μm. Arrows indicate autophagic bodies. **d** Confocal analysis showing co-localization of VISP1-GFP, 2b-mCherry, and CFP-NbATG8f in *N. benthamiana* leaves. VISP1^mUIM^-GFP served as a negative control. The infiltrated leaves were treated with 100 µM E64d or DMSO at 48 hpi and examined at 60 hpi. Arrows indicate autophagic bodies. Scale bars, 20 μm. Representative results of three independent experiments are shown. **e** Co-IP examining in vivo interactions between 2b-Flag and VISP1-GFP/VISP1^mUIM^-GFP/VISP1^mARM^-GFP. *N. benthamiana* leaves were infiltrated and treated with 1 mM 3-MA at 48 hpi, and collected for IP with anti-Flag beads at 64 hpi. Free GFP served as a negative control. **f** GST pull-down detecting in vitro interactions of 2b^N61^ with VISP1, VISP1^mUIM^, or VISP1^mAIM^. GST-VISP1, GST-VISP1^mUIM^, or GST-VISP1^mAIM^ were incubated with 2b^N61^-His and anti-GST beads for IP, and examined by immunoblotting analyses with anti-His or -GST antibodies. The sizes corresponding to the monomer, dimer, and tetramer forms of 2b^N61^-His were indicated. All experiments were repeated three times and similar results are provided in the Source Data file.

We have identified VISP1 as an autophagy receptor to trigger autophagic degradation of plant SGS3/RDR6 bodies for virus benefit^7^. Since VISP1 is induced by CMV infection, we inquired whether viral proteins were substrate cargoes of VISP1. To test this hypothesis, we carried out bimolecular fluorescence complementation assays (BiFC) to explore VISP1-interacting viral factors. The coding sequences of the CMV polymerase genes *1a*, *2a*, *movement protein (MP)*, *coat protein (CP)*, and *2b* genes were fused to the YFP N halve (Y^N^), and co-expressed with VISP1-Y^C^ in *N. benthamiana* leaves. At 60 h post-infiltration (hpi), intense YFP fluorescence was reconstituted in the cytoplasm and nucleus of leaf cells co-expressing VISP1-Y^C^ and 2b-Y^N^ proteins. By contrast, co-expression of VISP1-Y^C^ and CP-Y^N^ produced very faint YFP fluorescence, and other combinations failed to produce BiFC signal (Fig. 1a). Immunoblotting analyses verified expression of all proteins in the BiFC assays (Supplementary Fig. 1a, b). We have previously showed that VISP1 contains a ubiquitin-interacting motif (UIM, ^39^IISALTPS^44^) and an arginine/lysine-rich motif (ARM, ^28^RKLVK^32^) to interact with ATG8s and substrate proteins, respectively (Supplementary Fig. 2)^7^. Co-expression of 2b-Y^N^ with VISP1-Y^C^ or VISP1^mUIM^-Y^C^ produced YFP signal in the cytoplasm and nucleus, whereas co-expression of 2b-Y^N^ and VISP1^mARM^-Y^C^ failed to produce YFP signal (Fig. 1b), indicating that the VISP1 ARM domain is responsible for the interaction with CMV 2b.

We next examined whether CMV 2b was an autophagic cargo of VISP1. To this end, 2b-Y^N^ and VISP1-Y^C^ were co-expressed with CFP-NbATG8f in *N. benthamiana* leaves. Very few granules reconstituted by 2b-Y^N^/VISP1-Y^C^ were observed in DMSO-treated samples probably due to autophagic degradation (Fig. 1c). While, a significantly increased numbers of YFP granules were co-localized with CFP-NbATG8f-labeled autophagosomes in the leaves treated with E64d (Fig. 1c and Supplementary Fig. 1c). By contrast, 2b-Y^N^/VISP1^mUIM^-Y^C^ could not formed autophagosome structures because VISP1^mUIM^ could not interact with ATG8 proteins (Fig. 1c).

We further determined co-localization of VISP1, 2b, and NbATG8f in vivo through co-infiltration in *N. benthamiana* leaves. At 60 hpi, we observed co-localization of VISP1-GFP, 2b-mCherry, and CFP-NbATG8f in small granules in the leaves treated with E64d, but rarely in DMSO-treated leaves (Fig. 1d and Supplementary Fig. 1d). By contrast, no autophagosome structures were observed in leaves co-expressing VISP1^mUIM^-GFP, 2b-mCherry, and CFP-NbATG8f (Fig. 1d). These results suggest that VISP1 can drive CMV 2b into ATG8-anchored autophagosomes.

We next performed co-immunoprecipitation (Co-IP) assays to examine the VISP1–2b interaction in vivo. The 2b-Flag fusion protein was co-expressed with VISP1-GFP, VISP1^mUIM^-GFP, VISP1^mARM^-GFP, or GFP in *N. benthamiana* leaves. We treated infiltrated leaves with 1 mM 3-MA inhibitor at 48 hpi and collected for IP with anti-Flag affinity gels. The immunoblotting results showed that VISP1-GFP and VISP1^mUIM^-GFP, rather than VISP1^mARM^-GFP or GFP, were co-immunoprecipitated with 2b-Flag (Fig. 1e). We further examined VISP1–2b interaction using in vitro GST pull-down assays. Previous studies have shown that the full-length 2b protein is difficult to be purified from *Escherichia coli* (*E. coli*)^41^. Thus, the 2b truncated form harboring the N terminal 61 amino acid (2b^N61^) was fused with the 6×His tag and purified. The 2b^N61^-His protein was incubated with free GST, GST-VISP1, GST-VISP1^mUIM^, or GST-VISP1^mARM^ for immunoprecipitation with anti-GST beads. The immunoblotting analysis revealed that 2b^N61^-His was co-precipitated with GST-VISP1 and GST-VISP1^mUIM^, but not with GST-VISP1^mARM^ or GST (Fig. 1f). It is interestingly noted that the tetramer form of 2b^N61^-His was efficiently precipitated with GST-VISP1, although the monomer amount of 2b^N61^-His was much more than that of tetramer in input samples (Fig. 1f). Collectively, these results demonstrate that CMV 2b, mainly in tetramer forms, interacts with the ARM motif of VISP1.

### VISP1 mediates autophagic degradation of CMV 2b and restrains its VSR activity

**Fig. 2:**
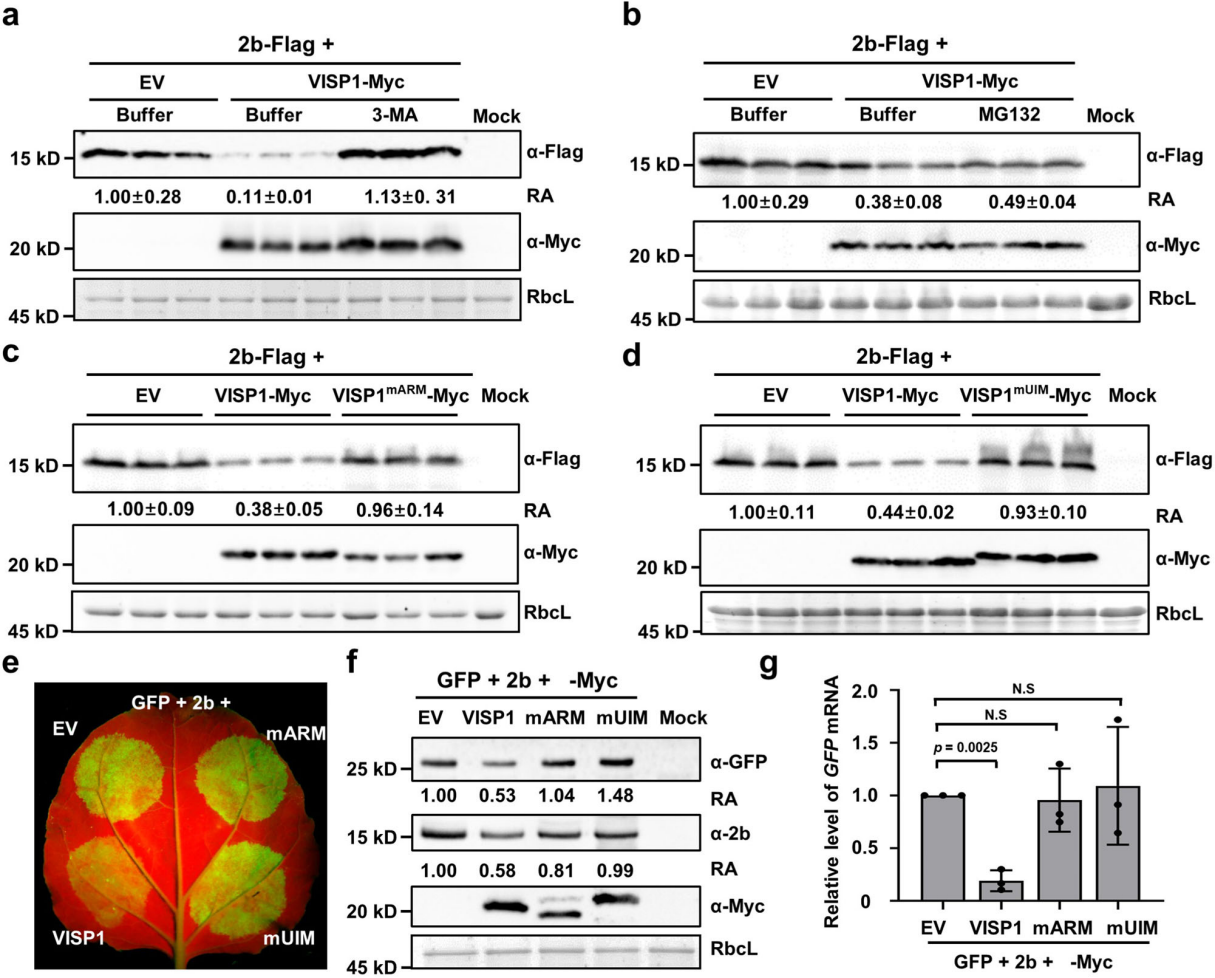
VISP1 mediates autophagic degradation of 2b and attenuates its VSR activity. **a** Effect of VISP1-Myc on accumulation of 2b-Flag in *N. benthamiana* leaves treated with buffer or 10 mM 3-MA at 48 hpi and harvested for immunoblotting analyses at 60 hpi. **b** Effects of VISP1-Myc and proteasome inhibitor MG132 on accumulation of 2b-Flag in *N. benthamiana* leaves treated with buffer or 100 µM MG132 at 48 hpi and collected for immunoblotting analyses at 60 hpi. **c** Immunoblotting analyses detecting accumulation of 2b-Flag co-expressed with VISP1-Myc or VISP1^mARM^-Myc in *N. benthamiana* leaves. **d** Immunoblotting analyses detecting accumulation of 2b-Flag co-expressed with VISP1-Myc or VISP1^mUIM^-Myc in *N. benthamiana* leaves. **e** GFP fluorescence in regions of *N. benthamiana* leaves co-expressing GFP, CMV 2b, and VISP1/VISP1^mARM^/VISP1^mUIM^/EV. The infiltrated leaves were photographed with a long-wave UV light at 5 dpi. **f** Immunoblotting analyses showing accumulation of GFP, 2b, and VISP1 in the samples of (**e**). **g** RT-qPCR analyzing accumulation of *GFP* mRNAs in the leaves of (**e**). Error bars indicate the mean ± SD of three biologically independent experiments. *EF1a* served as an internal reference. The *p* values by one-sided unpaired Student’s *t* test are indicated in the figures and the source data. Data information: EV, empty vector. **a**–**d** The relative accumulation (RA) values represent means ± SD calculated from band densities in three biological repeats. The RA values in panel (**f**) were calculated from band densities. (**a**–**d**, **f**) RbcL served as loading controls. The values of EV samples were set as 1. All experiments were repeated three times with similar results in the Source Data file.

We next determined whether VISP1 could mediate degradation of CMV 2b via autophagy pathway. The 2b-Flag protein was expressed with empty vector (EV) or VISP1-Myc in *N. benthamiana* leaves. At 60 hpi, we performed immunoblotting analyses and found that 2b-Flag accumulation was reduced to ~0.11-fold in the presence of VISP1-Myc compared to EV (Fig. 2a). Upon treatment with 10 mM 3-MA, 2b-Flag accumulation was recovered to about 1.13-fold of EV-treated leaves (Fig. 2a). By contrast, MG132 treatment did not obviously affect VISP1-induced reduction of 2b-Flag accumulation (Fig. 2b). Consistently, E64d (100 µM) and ConA (1 µM) also inhibited VISP1-mediated autophagic degradation of 2b-Flag (Supplementary Fig. 3a, b). We further knocked down expression of *NbATG5* and *NbATG7* using the TRV-induced gene silencing vectors, which have been used to knockdown the autophagy pathways^16^. The results showed that VISP1-mediated degradation of 2b-Flag was abolished in the leaves with silencing of *NbATG5* and *NbATG7* (Supplementary Fig. 3c).

Neither VISP1^mARM^ nor VISP1^mUIM^ affected 2b-Flag accumulation due to their defective interactions with 2b and ATG8s, respectively (Fig. 2c, d). In addition, accumulation of the CMV CP or MP proteins was not affected by co-expression of VISP1 because of their weak or negative interactions with VISP1 (Supplementary Fig. 4a, b). We have previously showed that the 2blm point mutant only form monomer and dimer, but not tetramer structures^41^. Since VISP1 mainly interacts with the tetramer form of 2b (Fig. 1f), we consistently showed that accumulation of 2blm-Flag, unlike that of 2b-Flag, was not obviously inhibited by co-expression of VISP1-Myc (Supplementary Fig. 4c). Collectively, these results indicate that CMV 2b is a cargo of VISP1 in selective autophagy.

CMV 2b is a well-known VSR that is usually examined by suppressing *GFP* silencing in co-infiltration assays^44^. In *N. benthamiana* leaves, green fluorescence of GFP transient expression almost disappeared at 5 dpi because of potent RNA silencing, whereas highly intense GFP fluorescence maintained in the leaf patches co-expressing GFP and 2b due to 2b-suppressed RNA silencing (Supplementary Fig. 5a). Co-expression of VISP1, but not VISP1^mARM^ or VISP1^mUIM^, significantly down-regulated 2b-mediated enhancement of GFP fluorescence compared to EV (Fig. 2e). Immunoblotting analysis consistently showed that VISP1 caused a reduced accumulation of the GFP and 2b proteins when compared to EV, VISP1^mARM^ or VISP1^mUIM^ (Fig. 2f). Reverse transcription quantitative PCR (RT-qPCR) assays showed that *GFP* transcript accumulated to a relative lower level in VISP1-coexpressing samples than other combinations (Fig. 2g).

Collectively, these results demonstrate that VISP1 mediates autophagic degradation of CMV 2b and attenuates its suppression activity on *GFP* silencing. Furthermore, we found that VISP1 could also mediate degradation of PoLV-encoded P14 and suppress its suppressor activity (Supplementary Fig. 5b). By contrast, VISP1 did not exhibit obvious effect on accumulation and suppressor activities of tomato bushy stunt virus (TBSV) P19 and barley strip mosaic virus (BSMV) γb proteins (Supplementary Fig. 5c, d).

### Knocking out of VISP1 facilitates late infection of wild-type CMV

We have previously obtained two CRISPR/Cas9-induced deletion mutants (*visp1-1* and *visp1-2*) within the *VISP1* ORF region. To confirm the deleted mutants, we analyzed accumulation of endogenous VISP1 using specific antibodies of VISP1 and could not detect accumulation of VISP1 in *visp1-1* mutants (Supplementary Fig. 6). By contrast, we detected low accumulation of endogenous VISP1 in Col-0 plants but was obviously induced by CMV infection at 7 dpi (Supplementary Fig. 6).

To examine the function of VISP1 in different virus infections, we inoculated Col-0, *visp1* mutants with wild-type CMV or CMV-2blm^41^. Plant viruses usually establish early infection at about 3 dpi in inoculated leaves. Subsequently, plant viruses establish extensive infection in systemically infected leaves at 10 dpi and 42 dpi, which is termed as late infection. In this study, we analyzed accumulation of viral proteins and RNAs at 3 dpi and 10/42 dpi to compare virus–plant interactions at different infection stages.

**Fig. 3:**
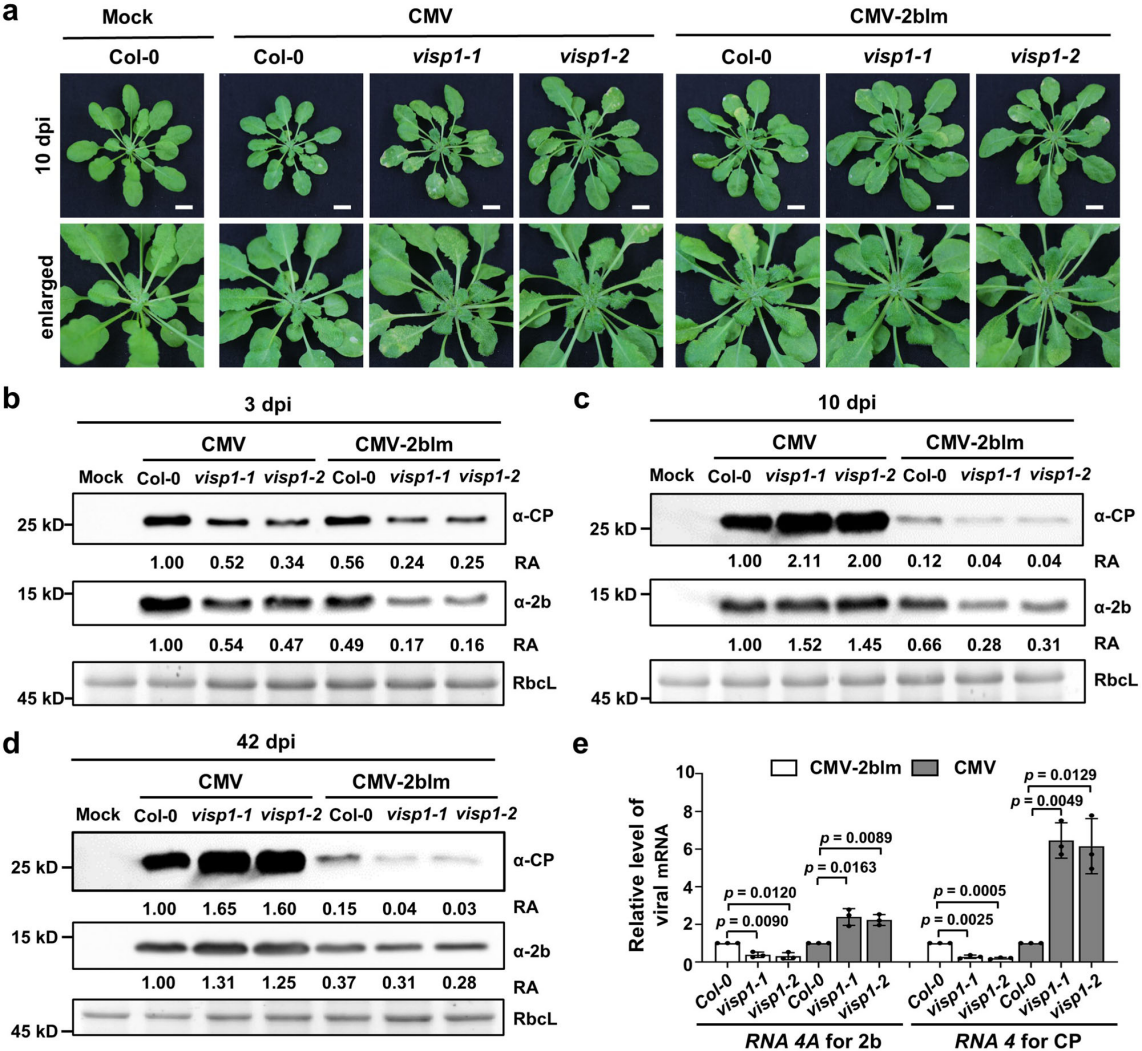
Knocking out of VISP1 facilitates late infection of wild-type CMV. **a** Symptoms of Col-0, *visp1-1*, and *visp1-2* plants inoculated with wild-type CMV and CMV-2blm at 10 dpi. Mock-treated Col-0 served as a negative control. Scale bars, 1 cm. **b** Immunoblotting analyses detecting accumulation of CMV CP and 2b proteins in Col-0, *visp1-1*, and *visp1-2* leaves inoculated with wild-type CMV and CMV-2blm at 3 dpi. **c** Immunoblotting analyses detecting accumulation of CMV CP and 2b proteins in Col-0, *visp1-1*, and *visp1-2* systemically infected leaves by wild-type CMV and CMV-2blm at 10 dpi. **d** Immunoblotting analyses detecting accumulation of CMV CP and 2b proteins in Col-0, *visp1-1*, and *visp1-2* systemically infected leaves by wild-type CMV and CMV-2blm at 42 dpi. The experiments were repeated three times with similar results. **e** RT-qPCR analyzing accumulation of *RNA4A* and *RNA4* of CMV in leaves of (**d**). The data represent the mean ± SD (*n* = 3). *Actin2* served as an internal reference. *** *p* < 0.001, ** *p* < 0.01, and * *p* < 0.05 vs. control (one-sided unpaired Student’s *t* test). In (**b**), (**c**), and (**d**), the relative accumulation (RA) values were calculated from band densities. RbcL served as loading controls. The values of EV samples were set as 1. Repeats with similar results are provided in a Source Data file.

In agreement with our previous results^7^, both *visp1-1* and *visp1-2* mutants exhibited enhanced resistance against CMV-2blm at 10 dpi (Fig. 3a). Immunoblotting analyses showed that accumulation of CP and 2b proteins was lower in *visp1-1* and *visp1-2* than in Col-0 plants at 3-, 10-, and 42 dpi (Fig. 3b–d). Besides, RT-qPCR results indicated that the CMV subgenomic *RNA4A* and *RNA4*, individually encoding 2b and CP proteins, accumulated to relative lower levels in *visp1* mutants compared with Col-0 plants (Fig. 3e). Because 2blm is VSR-deficient mutant^41^ and could not be targeted by VISP1 efficiently (Supplementary Fig. 4), VISP1 only triggered degradation of SGS3/RDR6 bodies as our previous studies^7^. Therefore, VISP1 only interferes with RNA silencing but not with virus pathogenicity, thereby facilitating CMV-2blm infection at both early (3 dpi) and late stages (10- and 42 dpi) of infection.

We next analyzed response of Col-0 and *visp1* mutants to wild-type CMV infection. Note that viral protein and RNA accumulation of CMV were significantly higher than those of CMV-2blm (Fig. 3c–e), because CMV 2b is a pathogenesis effector as described previously^32–35^. At 3 dpi, viral CP and 2b proteins accumulated to lower levels in *visp1* mutants compared to Col-0 (Fig. 3c), indicating that VISP1 facilitates CMV at early stage probably through degrading SGS3/RDR6 bodies. Subsequently, however, *visp1* mutants allowed higher levels of viral CP and 2b proteins at 10- and 42 dpi (Fig. 3c, d), as well as their RNA levels (Fig. 3e) at 42 dpi compared to Col-0 plants. The *visp1* mutants infected by CMV appeared more severely distorted leaf symptoms than Col-0 plants at 10 dpi (Fig. 3a). Consistently, the *visp1* mutants exhibited more dwarf symptoms than Col-0 plants at 42 days post infection of CMV (Supplementary Fig. 7).

These results indicate that VISP1 exhibits pro-viral activity in early infection (3 dpi) but changes reversely to antiviral activity in late infection (10- and 42 dpi). Collectively, these results indicate that VISP1-mediated 2b degradation contributes to its antiviral activity in virus late infection.

### Overexpression of VISP1 inhibits late infection of CMV

**Fig. 4:**
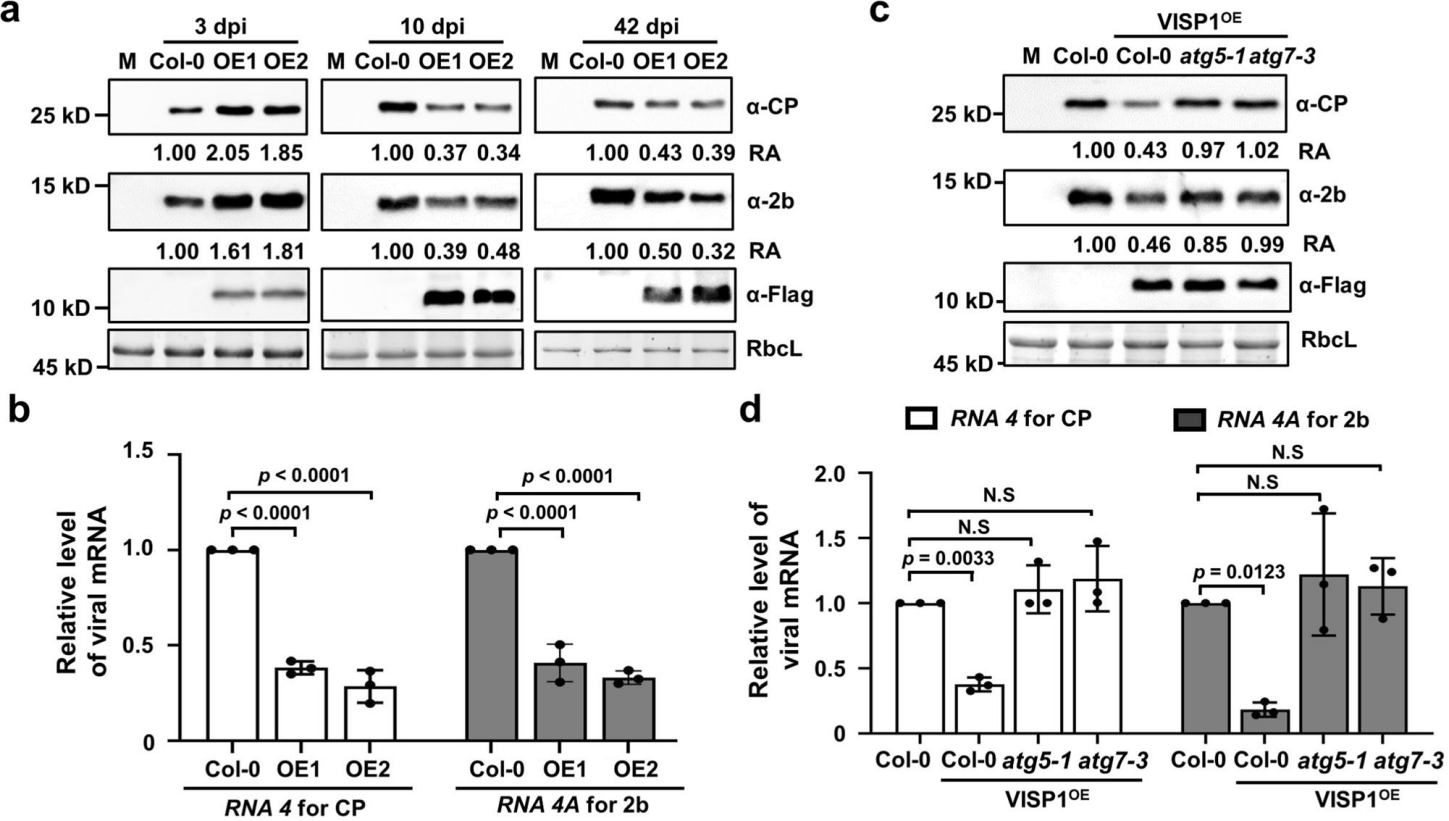
Overexpression of VISP1 inhibits late infection of wild-type CMV. **a** Immunoblotting analyses detecting accumulation of CMV CP, 2b proteins, and VISP1-Flag in Col-0, two independent VISP1-Flag transgenic plants (OE1 and OE2) inoculated with wild-type CMV at 3-, 10-, and 42 dpi. Note that total protein samples of 10-, and 42 dpi were diluted threefold and tenfold for loading and detection. M represents mock-treated Col-0. **b** RT-qPCR analyzing accumulation of *RNA4A* and *RNA4* of CMV in systemically infected leaves inoculated by CMV at 42 dpi in the (**a**). **c** Immunoblotting analyses detecting accumulation of CMV CP and 2b proteins, as well as VISP1-Flag in Col-0 and VISP1 overexpression lines in the background of Col-0, *atg5-1*, and *atg7-3* plants at 10 dpi. The experiments were repeated three times with similar results. **d** RT-qPCR analyzing accumulation of *RNA4A* and *RNA4* of CMV in systemically infected leaves of (**c**). In (**a**) and (**c**), the relative accumulation (RA) values were calculated from band densities. RbcL served as loading controls. The values of EV samples were set as 1. In (**b**) and (**d**), *Actin2* served as internal reference. The data represent the mean ± SD (*n* = 3 biological experiments). and the *p* values by one-way ANOVA (Dunnett’s test) are indicated. Repeats with similar results are provided in the Source Data file.

We next examined whether overexpression of VISP1 could inhibit CMV infection. To this end, we inoculated wild-type CMV on two independently transgenic Arabidopsis plants overexpressing VISP1-Flag (VISP1^OE1^ and VISP1^OE2^). At 3 dpi, both VISP1^OE1^ and VISP1^OE2^ plants allowed increased levels of CMV CP and 2b proteins compared to Col-0 plants (Fig. 4a, left panels). By contrast, two overexpression lines exhibited enhanced immunity against wild-type CMV at 10- and 42 dpi because they accumulated obviously lower levels of the CP and 2b proteins than Col-0 plants (Fig. 4a, middle and right panels). At 42 dpi, accumulation of CMV *RNA4* and *RNA4A* consistently reduced compared with that of Col-0 plants (Fig. 4b). These results indicate that VISP1 exerts pro-viral activity at the early infection but turns to limiting CMV infection at late infection.

To determine selective autophagy was involved in VISP1-mediated immunity against CMV, we obtained overexpression plants of VISP1 in *atg5-1* or *atg7-3* background and inoculated them with CMV. The immunoblotting analyses revealed that overexpression of VISP1 in the *atg5-1* or *atg7-3* background allowed increased accumulation of CMV CP and 2b proteins than in VISP^OE^/Col-0 plants (Fig. 4c). RT-qPCR analyses showed that VISP1 overexpression down-regulated accumulation of *RNA4* and *RNA4A* in Col-0 but not in *atg5-1* or *atg7-3* mutant background (Fig. 4d). Collectively, these results demonstrate that VISP1 overexpression inhibits virus late infection via autophagy pathway.

### VISP1 induces symptom recovery by triggering turnover of CMV 2b

**Fig. 5:**
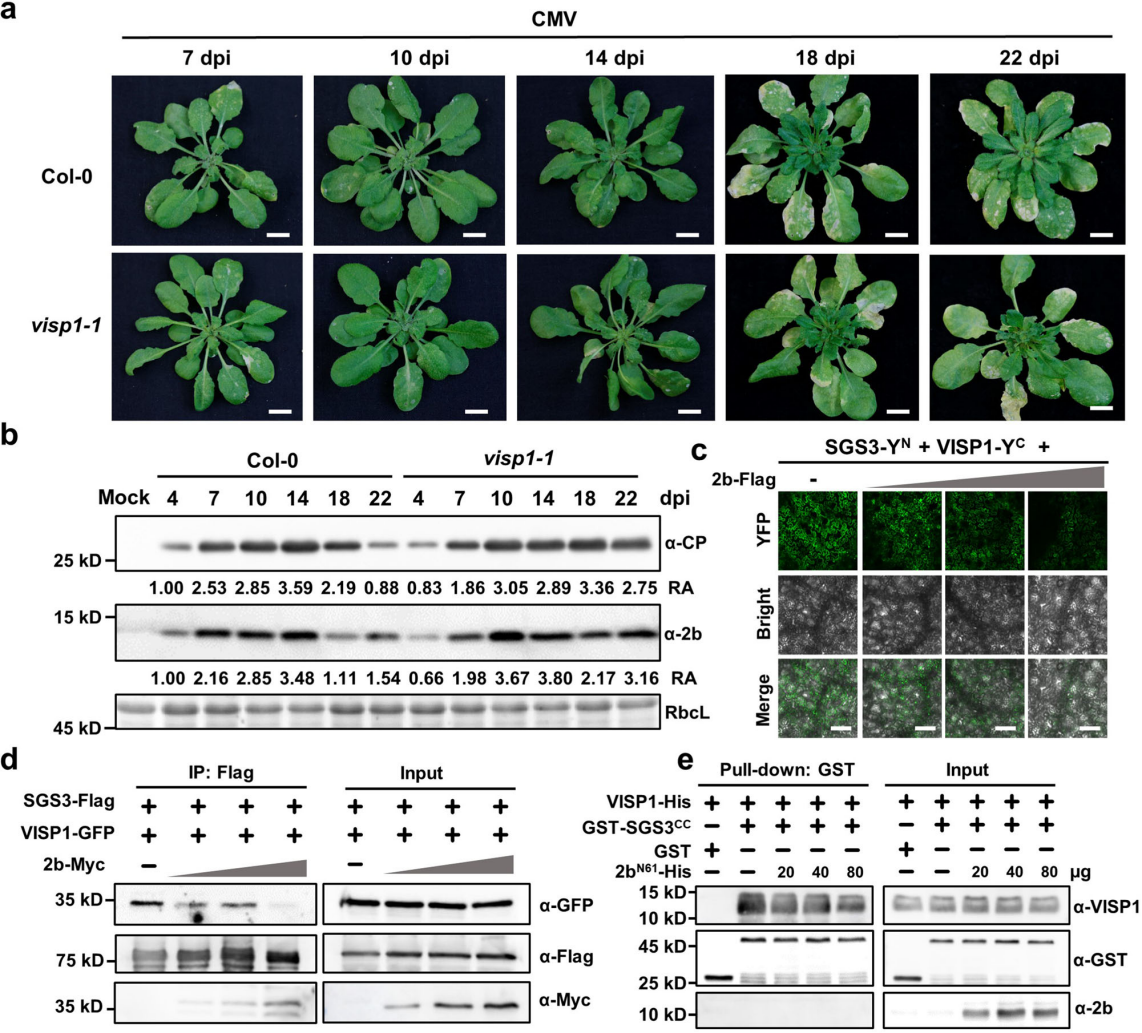
The increasing 2b protein along with virus infection compete for VISP1 with SGS3 for autophagic degradation. **a** Pathogenetic responses of Col-0 and *visp1-1* plants challenged with wild-type CMV at 7-, 10-, 14-, 18-, and 22 dpi. Scale bar, 1 cm. **b** Immunoblotting analyses detecting accumulation of CMV CP and 2b proteins in systemically infected leaves of plants in (**a**). RbcL served as loading controls. The experiments were repeated three times with similar results. **c** Competitive BiFC assays examining the effect of 2b on the VISP1–SGS3 interaction. The plasmids expressing SGS3-Y^N^ (OD_600_ = 0.2) and VISP1-Y^C^ (OD_600_ = 0.2) were co-infiltrated with increasing concentrations (OD_600_ = 0, 0.02, 0.1, 0.5 of 2b-Flag. Images were taken at 60 hpi. Scale bars, 100 μm. The experiments were repeated three times with similar results. **d** Co-IP assays showing the competitive effect of 2b on the VISP1–SGS3 interaction. SGS3-3×Flag and VISP1-GFP were co-expressed with increasing concentrations of 2b-Myc in *N. benthamiana* leaves. After treated with 1 mM 3-MA at 48 hpi, the leaves were collected for IP with anti-Flag beads 16 h later. **e** GST-pull down assays examining the competitive effect of 2b on the VISP1–SGS3 interaction in vitro. GST-SGS3^CC^ (20 μg), VISP1-His (20 μg), and increasing amount of 2b^N61^-His (20-, 40-, 80 μg) were immunoprecipitated with anti-GST beads, and examined by immunoblotting analyses with anti-His or -GST antibodies. The experiments were repeated two times with similar results. Source data are provided as the Source Data file.

In viral early infection, VISP1 targets SGS3/RDR6 bodies as degradation cargos, resulting in compromised RNA silencing and enhanced virus infection. However, it can be reasonably assumed that amount of 2b gradually increased along with virus infection and competed with SGS3/RDR6 for VISP1 to be degraded in autophagy pathway, leading to VISP1-mediated inhibition of CMV late infection. Indeed, time-course infection assays showed that CMV induced severe symptoms at 14- and 18 dpi, but the symptoms appeared recovery at 22 dpi in Col-0 plants (Fig. 5a). By contrast, the severe symptoms of *visp1-1* mutants still maintained at 22 dpi, indicating a positive role of VISP1 in symptom recovery (Fig. 5a). Immunoblotting analyses consistently revealed that accumulation of CMV CP and 2b increased to a high level at 14 dpi and then decreased gradually at 18- and 22-dpi in Col-0 plants (Fig. 5b, left panels). By contrast, both CP and 2b gradually accumulated to high levels and did not decrease in *visp1-1* mutants at 14-, 18-, and 22 dpi (Fig. 5b). These results imply that VISP1-mediated 2b degradation is a brake factor to induce symptom recovery from severe infection.

Since both CMV 2b and SGS3 interact with the same ARM domain of VISP1 (Fig. 1 and Supplementary Fig. 2)^7^, CMV 2b would compete with SGS3 for VISP1 in a dose dependent manner. Accordingly, we carried out competitive BiFC assays to determine whether increasing amount of CMV 2b could interfere with the VISP1–SGS3 interaction. As expected, we found that BiFC fluorescence signal of SGS3-Y^N^ and VISP1-Y^C^ gradually decreased along with increased accumulation of 2b-Flag (Fig. 5c and Supplementary Fig. 8). We further performed Co-IP assays to confirm their competence for VISP1. To this end, we infiltrated Agrobacterium cultures for expression of SGS3-Flag and VISP1-GFP along with increasing concentration of 2b-Myc in *N. benthamiana* leaves. The infiltrated leaves were treated with 1 mM 3-MA at 48 hpi and then collected for IP with anti-Flag affinity gels at 64 hpi. Immunoblotting analyses showed that gradually decreased amount of VISP1-GFP was immunoprecipitated with SGS3-Flag along with increased levels of 2b-Myc (Fig. 5d). In vitro GST pull down assays consistently showed that increasing amount of 2b^N61^-His protein suppressed co-precipitation of VISP1-His with GST-SGS3^CC^ (Fig. 5e). Collectively, these results demonstrate that VISP1 targets increasing amount of CMV 2b instead of SGS3 for autophagic degradation and consequently exhibits antiviral activity in CMV late infection.

### VISP1 mediates autophagic degradation of the C2/AC2 VSRs of two geminiviruses and inhibits virus infections

In addition to RNA viruses, plant-infecting DNA viruses encode distinct VSRs to counter-defense against RNA silencing. Geminiviruses are single-stranded DNA viruses composing the largest family of plant viruses. The geminivirus-encoded multifunctional AC2/C2 proteins are transcription activator proteins (TrAPs) functioning in activation of late viral genes and suppress host defense^45,46^. The C2 protein (C2^BSCTV^) of BSCTV and the AC2 (AC2^CaLCuV^) protein of CaLCuV were selected for VISP1 degradation analysis, because BSCTV and CaLCuV can infect Arabidopsis and be used for genetic analyses in VISP1 overexpression and knock-down plants.

**Fig. 6:**
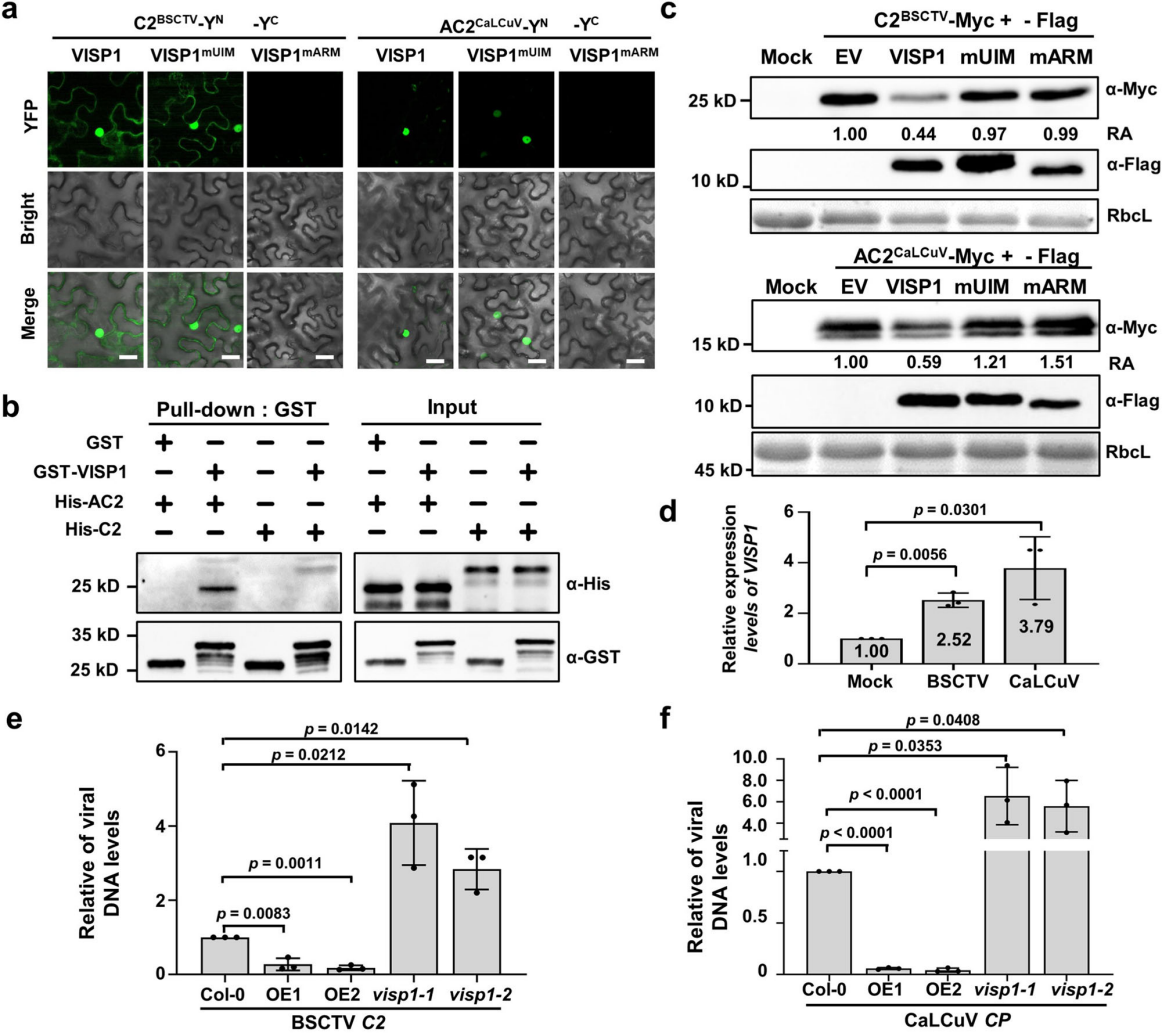
VISP1 mediates autophagic degradation of the C2/AC2 VSRs of two geminiviruses and inhibits virus infections. **a** BiFC analyses of interactions between VISP1 and C2^BSCTV^/AC2^CalCuV^ proteins. Infiltrated *N. benthamiana* leaves were photographed at 60 hpi. Scale bar = 50 μm. **b** GST pull-down detecting in vitro interactions of VISP1 with C2^BSCTV^ or AC2^CalCuV^. GST-VISP1, or GST were incubated with His-C2^BSCTV^ or His-AC2^CalCuV^ and anti-GST beads for pull-down, and examined by immunoblotting analyses with anti-His or -GST antibodies. **c** Immunoblotting analyses detecting accumulation of C2^BSCTV^-Myc or AC2^CaLCuV^-Myc co-expressed with VISP1-Flag, VISP1^mUIM^-Flag, or VISP1^mARM^-Flag in *N. benthamiana* leaves. **d** RT-qPCR analyses of relative *VISP1* mRNA accumulation in systemically infected leaves by mock buffer, BSCTV, and CaLCuV at 14 dpi. The value in mock-treated plants were set as one unit. **e** and **f** Relative viral titer of BSCTV and CaLCuV in Col-0, VISP1^OE^ (OE1 and OE2), and *visp1-1/1-2* plants at 14 dpi. Quantitative PCR was performed using the specific primers corresponding to BSCTV *C2* or CaLCuV *CP* genes. *Actin2* as an internal genomic DNA control. Viral titers in Col-0 plants were set as one unit. In (**d**), (**e**), and (**f**), the data represent the mean ± SD (*n* = 3). The *p* values by one-sided unpaired Student’s *t* test are indicated. All experiments were repeated three times biologically. Source data are provided as the Source Data file.

We first used BiFC analyses to show that both C2^BSCTV^ and AC2^CaLCuV^ could interact with VISP1 and VISP1^mUIM^, but not with VISP1^mARM^ (Fig. 6a). Moreover, most of these BiFC signal was observed in the nucleus and very faintly in the cytoplasm (Fig. 6a). Immunoblotting analyses verified expression of all proteins in the BiFC assays (Supplementary Fig. 9a, b). Moreover, the E64d treatment increased numbers of BiFC-labeled granules, which were co-localized with CFP-NbATG8f in mesophyll cells (Supplementary Fig. 9c–f). We further carried out GST Pull-down assays to examine their direct interactions. Immunoblotting analyses showed that His-AC2 and His-C2 could be immunoprecipitated with GST-VISP1, rather than GST (Fig. 6b). Collectively, the results demonstrate the interactions of C2^BSCTV^/AC2^CaLCuV^ and VISP1 in vivo and in vitro.

We next determined whether VISP1 targeted C2^BSCTV^ and AC2^CaLCuV^ for autophagic degradation. C2^BSCTV^-Myc or AC2^CaLCuV^-Myc was co-expressed with EV, VISP1, VISP1^mUIM^, or VISP1^mARM^ in *N. benthamiana* leaves. At 3 dpi, immunoblotting analyses revealed that co-expression of VISP1 significantly down-regulated accumulation of the C2^BSCTV^-Myc and AC2^CaLCuV^-Myc proteins (Fig. 6c). By contrast, neither VISP1^mUIM^ nor VISP1^mARM^ exhibited obvious effects on accumulation of C2^BSCTV^-Myc or AC2^CaLCuV^-Myc (Fig. 6c). Moreover, the E64d treatment could inhibit VISP1-mediated autophagic degradation of C2^BSCTV^-Myc and AC2^CaLCuV^-Myc (Supplementary Fig. 10).

To examine the genetic functions of VISP1 in virus infections, we inoculated Col-0, VISP1^OE1^, VISP1^OE2^, *visp1-1*, and *visp1-2* plants with BSCTV or CaLCuV. At 14 dpi, BSCTV and CaLCuV infections increased accumulation of *VISP1* to 2.70- and 3.72-fold of mock buffer treated Col-0 plants (Fig. 6d). At 14 dpi, systemically infected leaves were collected for qPCR analyses using the primers corresponding the BSCTV *C2* and CaLCuV *CP* regions, respectively. The results showed that both BSCTV and CaLCuV accumulated significantly lower levels in two VISP1 overexpression lines, but increased in two *visp1* mutants when compared with Col-0 plants (Fig. 6e, f). At 42 dpi, VISP1 also inhibit infections of BSCTV and CaLCuV in the symptom development and virus accumulation (Supplementary Fig. 11). In contrast, VISP1 exhibited pro-viral activity in inoculated leaves at 3 dpi (Supplementary Fig. 12), which was in consistence with early infection of CMV (Figs. 3 and 4). Collectively, these results indicate that VISP1 can mediate degradation of the C2/AC2 proteins and thereby negatively regulate late infections of BSCTV and CaLCuV.

**Fig. 7:**
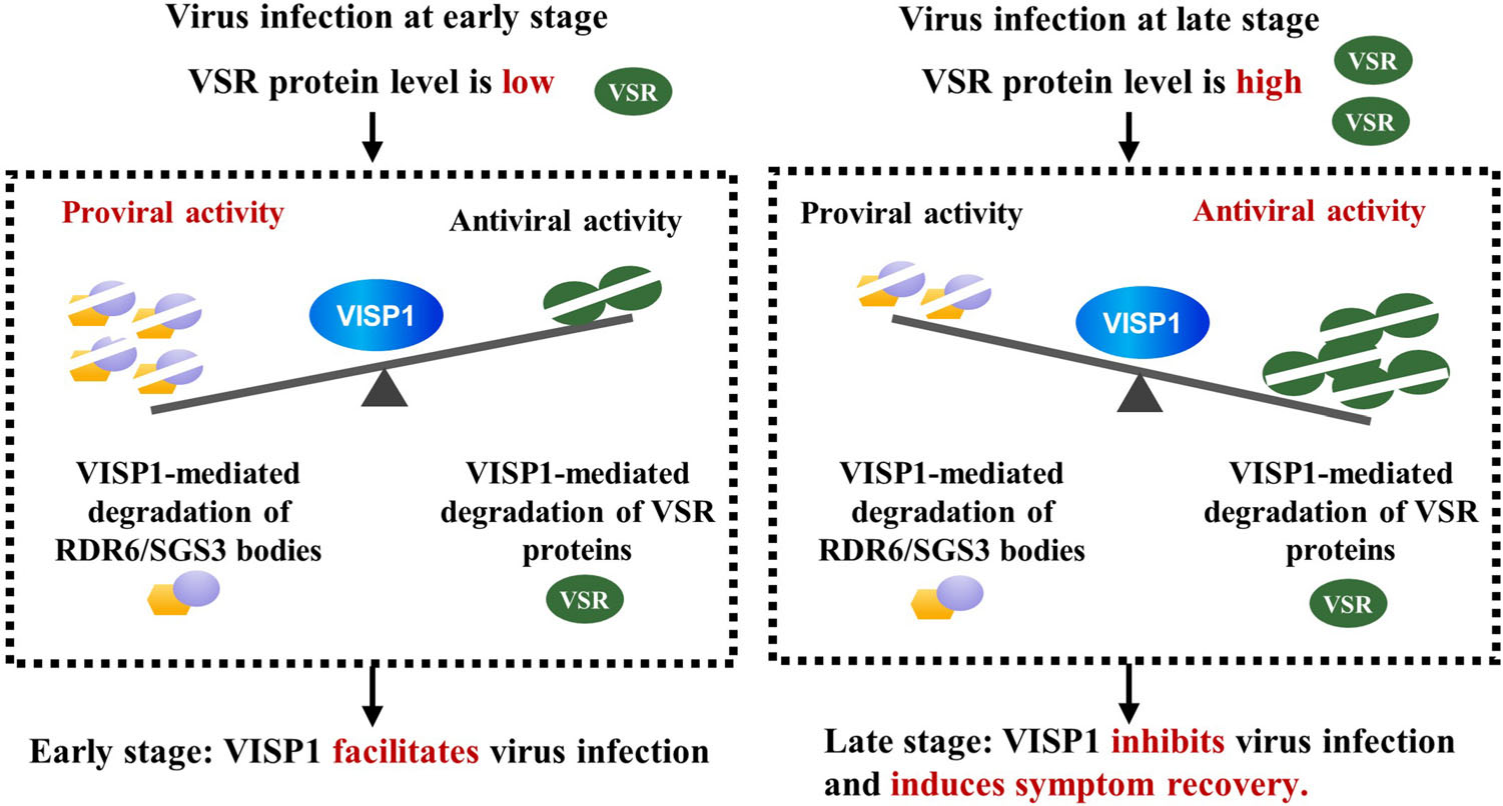
Model of VISP1-induced symptom recovery through balancing plant RNA silencing and viral suppressors of RNA silencing. The virus-induced VISP1 acting a selective autophagy receptor mediates autophagic degradation of SGS3/RDR6 bodies and viral suppressors of RNA silencing (VSRs). Since accumulation of VSRs was very low at early stages of virus infection, VISP1 mainly mediates degradation of SGS3/RDR6 bodies, thereby facilitating virus infection. Along with virus infection, amount of VSRs increase and compete for VISP1 with SGS3/RDR6 bodies, and become main substrates of VISP1-mediated autophagy, which resulted in antiviral activity of VISP1. Therefore, VISP1 upsets the balance of the arms race between host plants and viruses, thereby inducing symptom recovery.

In conclusion, we demonstrate that VISP1, acting as a selective autophagy receptor, targets VSRs of CMV, BSCTV, and CaLCuV for autophagic degradation. Along with virus infections, increasing amount of VSRs are the main targets of VISP1 in selective autophagy, resulting in autophagic degradation of VSRs and inducing symptom recovery from severe infections of plant viruses (Fig. 7). Our data provide clues for understanding defense, counter-defense, and balance relationship between viruses and host plants.

## Discussion

Selective autophagy is a powerful strategy in antiviral immunity of host plants. Conversely, plant viruses have evolved various strategies to subvert selective autophagy for successful infections^9,47^. The pro-viral or antiviral activities of autophagy are mainly determined by specific recognition between selective receptors and their substrates. We previously identified a virus-induced VISP1 as a selective receptor to subvert antiviral RNA silencing for virus benefit^7^. However, we here found that VISP1 inhibited virus infection through mediating turnover of the CMV-encoded 2b protein, a viral VSR and pathogen determinant effector. We previously used a 2b-deficient virus mutant CMV-2blm that cause weak symptoms in wild-type Col-0 plants^41^. Therefore, VISP1-mediated degradation of SGS3/RDR6 bodies lead to attenuated RNA silencing and enhanced CMV-2blm infection^7^. However, during wild-type CMV infection, VISP1 targets both SGS3/RDR6 bodies and CMV 2b, thereby balancing the pro-viral and antiviral activities. We found that VISP1 exhibited distinct roles at early and late stages of CMV infection (Fig. 4). Thus, we conclude that VISP1 is beneficial for virus early infection by down-regulating SGS3/RDR6-mediated siRNA amplification. Along with increasing accumulation of virus and 2b protein in the late infection, VISP1-induced degradation of 2b instead of SGS3/RDR6 bodies leads to suppression of virus replication speed, which is in turn benefit for plant integrity and virus long-term infection (Fig. 7). Therefore, VISP1 acting as a double-edged sword fine-tunes antiviral RNA silencing and virus pathogenesis.

Naturally, symptom recovery is commonly observed in shoot apices of infected plants, which represents final balance of the ongoing evolutionary “arms race” between host plants and viruses. However, how to reach the balance in infected plants is still being elucidated. The CMV 2b protein is a well-known VSR and an important virulence effector^32,33,35,41^. Therefore, symptom recovery could be achieved through controlling 2b-induced abnormal development and virus overwhelming accumulation. We have previously shown that CMV CP down-regulates accumulation of CMV 2b for viral self-attenuation and symptom recovery^48^. In addition, Nakahara et al. have demonstrated that the *Nicotiana tabacum* rgs-CaM protein (NtCaM) triggers autophagic degradation of CMV 2b and the potyvirus Hc-Pro protein, but the responsible autophagy receptors remain unknown^36^. However, other studies demonstrate that the rgs-CaM proteins indeed function as endogenous suppressors of RNA silencing and facilitates virus infection^21,49^. In Arabidopsis, CML39, a close homologue of tobacco rgs-CaM, was found to facilitate infection of tomato golden mosaic virus (TGMV) in genetic assays^50^. However, whether CML39 triggers autophagic degradation of SGS3 or CMV 2b remains to be verified in Arabidopsis. Here, our results clearly indicate that VISP1-mediated autophagy targets the 2b protein for degradation, a strategy to control accumulation of virulence determinant.

Geminiviridae is the largest family of plant viruses with more than 500 species infecting a great number of monocots and dicots worldwide^51^. The AC2 protein of begomoviruses and the C2 protein of curtoviruses suppress PTGS and TGS via transcription dependent and independent mechanisms^50,52–54^. However, how host factors counteract C2/AC2-mediated defense remains largely unknown. Although interacting with the tobacco rgs-CaM protein, the TGMV AL2 protein is not degraded probably because of the nuclear localization^50^. Here, we found that VISP1 targeted the CaLCuV AC2 protein and the BSCTV C2 for selective degradation. In addition, VISP1 exhibited antiviral immunity against infections of these two geminiviruses in Arabidopsis. Therefore, these results provide evidence showing defense, counter-defense, counter-counter-defense between host plants and plant viruses in an interesting zig-zag model of plant immunity^55^.

Substrate recognition by autophagy cargo receptors is an interesting but elusive question. The mammalian cargo receptor p62 is a ubiquitin-binding protein that specifically interacts with protein aggregates with ubiquitination modification for degradation^4^. Therefore, ubiquitination acts as an “eat me” signal decorating those unwanted substrates^56^. The proteasome usually degraded small ubiquitinated cargos due to the spatial capacity of proteasomes, whereas selective autophagy is harnessed to degrade protein aggregates decorated with “eat-me” signal^56^. In addition, other “eat me” signals or direct interaction might mediate cargo recognition. Here, we show that VISP1 only recognizes the VSR proteins rather than other viral proteins. Interestingly, both CMV 2b and PoLV P14 are size-independent dsRNA-binding VSRs, which can be targeted by VISP1^33,57^. Whereas, VISP1 has no effect on accumulation of TBSV P19 and BSMV γb that efficiently bind 21 nt ds-sRNA but fail to bind long dsRNA^58^. The results indicating that some “eat me” signals might be present in these VSRs substrates harboring size-independent dsRNA-binding activity. Further studies are needed to clarify whether some yet known “eat-me” signal on the cargo of VISP1 will be further explored.

In summary, VISP1, an autophagy receptor, targets multiple cargoes including VSRs of RNA and DNA viruses, as well as antiviral SGS3/RDR6 bodies. Our results reveal that VISP1-mediated autophagy fine-tune plant resistance and virus pathogenesis, modulating the ongoing “arms race” between host plants and viruses. The dual roles of VISP1 might be manipulated by viruses to facilitate virus early infection, but act as a brake to prevent deleterious effects of virus overwhelming replication. VISP1 thereafter controls plasticity of virus infection and enable infected plants to thrive for virus transmission by insect vectors. Our finding adds a layer to the zig-zag model among virus–host plants interaction, and enriches our knowledge in cross-talks of autophagy, RNA silencing, and plant immunity against pathogens.

## Methods

### Plant materials and growth conditions

All *Arabidopsis thaliana* (*A. thaliana*) plants are in the Columbia ecotype background. The VISP1 overexpression lines (VISP1^OE1^ and VISP1^OE2^) and *visp1* mutant plants (*visp1-1* and *visp1-2*), as well as overexpression lines of VISP1-Flag in *atg5-1* and *atg7-3* background^7^. *A. thaliana* seeds were plated on MS medium (Caisson Laboratories, Rexburg) with 3% sucrose, 1% agar, and pH 5.8. Seedlings were vernalized in dark at 4 °C for 3 days (d), and removed to a chamber at 22 °C for 7 d. Seedlings were transferred into soil in a growth room at 22–24 °C in a 10 h-light/14 h-dark cycle. *N. benthamiana* plants were grown in a room with a 16 h-light/8 h-dark cycle at 25 °C.

### Virus inoculation

CMV virions were diluted with buffer C (0.5 mM Na_2_EDTA, 5 mM sodium borate, pH 9.0) to required concentrations. Three expanded leaves of 4-week-old *A. thaliana* seedlings were inoculated with wild-type CMV (10 ng/μL) or CMV-2blm (30 ng/μL)^41,42^. The inoculated leaves at 3 or 4 dpi and systemically infected leaves at indicated dpi were collected for virus protein and RNA analyses. BSCTV inoculation was achieved by infiltrating *Agrobacterium tumefaciens* (*A. tumefaciens*) EHA105 cultures harboring the pCambia1300-BSCTV (OD_600_, 0.5) that was generated by introducing the full-length genomic DNA of BSCTV into the pCambia1300 vector. CaLCuV inoculation was performed with *A. tumefacien* EHA105 carrying both pCambia1300-CaLCuV-A (OD_600_, 0.5) and pCambia1300-CaLCuV-B (OD_600_, 0.5)^59,60^. All virus inoculation and analyses experiments were repeated at least three times with reproducible results.

### Bimolecular fluorescence complementation (BiFC) and confocal microscopy

BiFC assays were carried out as described previously^61^ with minor modification. The VISP1-Y^C^, VISP1^mUIM^-Y^C^, and VISP1^mARM^-Y^C^ plasmids have been described^7^. The ORFs of CMV genes including *1a*, *2a*, *MP*, *CP*, and *2b*, as well as different VSRs including *BSCTV C2* and *CaLCuV AC2* were cloned into the pSPYNE-35S vector^62^ and fused to the N-terminus of YFP (1-173, Y^N^). *A. tumefaciens* containing two BiFC plasmids and the pGD-P19 plasmid were co-infiltrated into *N. benthamiana* leaves at a ratio of 0.4:0.4:0.1 (OD_600_). After co-infiltrated by *A. tumefaciens* with AC2-Y^N^/C2-Y^N^/2b-Y^N^, VISP1-Y^C^/ VISP1^mUIM^-Y^C^, CFP-NbATG8f, and P19, leaves were treated with 100 μM E64d or DMSO at 48 hpi and examined at 60 hpi for autophagic bodies observation and analyses. YFP fluorescence of infiltrated leaves was observed using a Zeiss LSM 880 confocal microscope at 60 hpi. For co-localization of VISP1-GFP/VISP1^mUIM^-GFP, 2b-mCherry, and CFP-NbATG8f, the infiltrated *N. benthamiana* leaves were treated with 100 μM E64d or DMSO at 48 hpi. After a 12 h-treatment of dark, infiltrated leaves were examined at 60 hpi. Fluorescence of CFP, GFP, and mCherry were monitored with excitation wavelengths of 440-, 488-, and 561 nm, respectively. Chloroplast II auto-fluorescence was recorded with light emitted at 633 nm. All used specific primers were listed in Supplementary Data 1.

### Immunoblotting analyses

Immunoblotting analyses were performed as described previously^63^. Total proteins were extracted in 2×volume of SDS loading buffer [100 mM Tris-HCl (pH 6.8), 20% (v/v) glycerol, 0.2% (w/v) bromophenol blue, 4% (w/v) SDS, and 5% (v/v) β-mercaptoethanol]. After boiled for 10 min and centrifuged at 13,523 *g* for 10 min, supernatants were separated in SDS-PAGE gels (7.5–20%), and transferred to nitrocellulose membranes by wet transfer. Membranes were blocked with 5% (w/v) defatted milk powder in 10 mL TBST buffer [20 mM Tris-HCl (pH 7.5), 150 mM NaCl, 0.05% (v/v) Tween-20] for 1 h at 37 °C. All primary antibodies listed below were added to 10 mL TBST buffer with different ratio by incubation at 37 °C for 1 h or 4 °C overnight. The GFP, CMV CP, 2b, and GST-tagged proteins were detected using polyclonal antibodies anti-GFP (1:1000), anti-CP (1:3000), anti-2b (1:1000), and anti-GST (1:10,000) acquired from rabbits immunized with purified proteins. In addition, Flag-, Myc-, HA-, and His-tagged proteins were detected with anti-Flag (1:5000, Sigma Cat. No. F7425), anti-Myc (1:5000, EASYBIO, Cat. No. BE2011), anti-HA (1:10,000, MBL, Cat. No. M180-3), and anti-His (1:5000, Proteintech, Cat. No. 66005), respectively. After washed three times with TBST buffer, membranes were incubated with the goat anti-rabbit IgG (H + L) horseradish peroxidase conjugate (1:20000, EASYBIO, Cat. No. BE0101) or goat anti-mouse IgG (H + L) horseradish peroxidase conjugate (1:3000, Bio-Rad, Cat. No. 170-6516) for 45 min at 37 °C. Membranes were added chemiluminescent substrate and detected by Azure Biosystems C600 (Azure, America).

### In vivo co-immunoprecipitation (Co-IP) assays

Co-IP assays were performed as previously described^64^ with minor modification. The pGD-2b-3×Flag, pGDGm-VISP1-GFP, pGDGm-VISP1^mARM^-GFP, and pGDGm-VISP1^mUIM^-GFP have been described previously^7,41^. *N. benthamiana* leaves were agroinfiltrated with *A. tumefaciens* cultures containing the binary plasmids for expression 2b-3×Flag, VISP1-GFP, and P19 at a ratio of 0.5:0.5:0.1 (OD_600_). Infiltrated leaves were treated with 1 mM 3-MA at 48 hpi and harvested for IP at 60 hpi. Approximately 3 g infiltrated leaves were ground in liquid nitrogen and homogenized in extraction buffer [10% (v/v) glycerol, 50 mM Tris–HCl (pH 7.5), 100 mM NaCl, 1 mM EDTA, 0.1% (v/v) NP-40, 2% (w/v) PVP-40, 1% (v/v) cocktail, and 10 mM DTT] for 40 min on the ice, and centrifuged at 13,523 *g* for 40 min at 4 °C.After filtration with glass funnels and gauzes, supernatants were incubated with 4% (w/v) BSA blocked anti-Flag affinity gels (GNI, 4510-FG) on a rotational mixer (Kylin-Bell Lab Instruments, WH-986) for 4 h at 4 °C. The beads were washed three times with IP buffer [10% (v/v) glycerol, 50 mM Tris–HCl (pH 7.5), 200 mM NaCl, 1 mM EDTA, 0.1% (v/v) NP-40] for 10 min each time, and the IP products were examined using immunoblot analyses. For competitive Co-IP assays, increasing concentrations of pGD-2b-6×Myc (OD_600_ 0.02, 0.1, and 0.5) were agroinfiltrated with pMDC32-SGS3-3×Flag (OD_600_, 0.2), pGDGm-VISP1-GFP (OD_600_, 0.2), and P19 (OD_600_, 0.2). These tissues were harvested for IP at 60 hpi, and were ground to powder with pestles. The extraction method of plant proteins was same as above. After washed three times with IP buffer, samples were analyzed by immunoblotting with antibodies of anti-GFP, anti-Flag, and anti-Myc.

### GST pull-down assay

The GST-VISP1, GST-VISP1^mUIM^ and GST-VISP1^mARM^ proteins were purified as described previously^7^. The pET28a-2b^N61^ and pET28a-2blm^N61^ for expression the N-terminal 61 amino acids of CMV 2b or 2blm have been described previously^41^. The coding sequences of C2^BSCTV^ and AC2^CaLCuV^ were cloned into pET30a (+) vector to generate His-C2^BSCTV^ and His-AC2^CaLCuV^ clones. All recombinant plasmids were transformed into *E. coli* BL21 for protein expression and purification as described previously^7^. In binding assays, purified GST fusion proteins (20 µg) and prey proteins (20 µg) were incubated with anti-GST beads in 500 µL binding buffer [50 mM Tris–HCl pH 7.5, 100 mM NaCl, 0.6% (v/v) Triton X-100, 10 mM DTT, 0.2% (v/v) glycerol, and 1 mM PMSF] on a rotational mixer at room temperature for 2.5 h. After washed 5 times with 1 mL washing buffer [50 mM Tris–HCl (pH 7.5), 200 mM NaCl and 0.6% (v/v) TritonX-100], the beads were boiled in 60 µL washing buffer and 60 µL 2×SDS–PAGE loading buffer for 10 min. After centrifuged at 13,523 *g* for 2 min, the supernatants were subjected to SDS–PAGE analyzed by specific antibodies. For competitive pull-down assays, 20 μg, 40 μg, and 80 μg 2b^N61^-His protein were added in the mixtures of GST-VISP1 (20 μg) and SGS3^CC^-His (20 μg), followed by incubation with GST beads in binding buffer on a rotational mixer at room temperature for 2.5 h. After three washes with washing buffer, samples were analyzed by immunoblotting analysis with antibodies of anti-VISP1, anti-GST, and anti-2b.

### Suppression of local *GFP* silencing assays

For transient expression in *N. benthamiana* leaves, the cDNAs of CMV 2b, PoLV P14, BSMV γb, and TBSV P19 were engineered into the pGD binary vector^65^ to generate pGD-2b-3×Flag, pGD-P14-9×Flag, pGD-γb-3×Flag^66^, and pGD-P19-9×Flag clones. Then, the resultant plasmids transformed into the *A. tumefaciens* EHA105 strain. VSR detection was performed as previously described^48^. *N. benthamiana* leaves were co-infiltrated with *A. tumefaciens* cultures harboring plasmids expressing positive sense GFP (sGFP), pGD-VISP1-6Myc or its mutants and different VSRs in a ratio of 0.5:0.5:0.1 (OD_600_). At 5 dpi, GFP fluorescence of agroinfiltrated leaves was observed and photographed under a long wavelength UV lamp (UVP, Upland, CA, USA) using a 600D Canon digital camera with a yellow filter. Protein accumulation in local silencing experiments was examined with immunoblotting analyses.

### Preparation of anti-VISP1 antibodies for detection of endogenous VISP1

To generate the anti-VISP1 antibodies, the Arabidopsis *VISP1* cDNA was cloned into the pET30a (+) vector. The VISP1-His fragment was expressed and purified from *E. coli* with Ni-NTA agarose (QIAGEN). Then, the purified VISP1-His protein was used to immunize rabbits to obtain polyclonal antibodies (Beijing Genomics institution, China). The polyclonal antibodies of VISP1 (10 mL) were diluted with 40 mL PBS buffer (155 mM NaCl, 1 mM KH_2_PO_4_, 3 mM Na_2_HPO_4_, pH 7.4) incubated with 1 mL protein G agarose (Beyotime) on the rotational mixer for 4 h at 4 °C. After washed 5 times with PBS buffer, antibodies were eluted from the agarose beads with 100 mM glycine (pH 2.7 and 1.9, 9 mL each). After neutralized with 1 M Tris-HCl (pH 8.8), the products were concentrated to 1 mL as preliminary antibodies. For purify antibodies with antigen affinity, 2 mg VISP1-His proteins were boiled with 5×SDS-PAGE loading buffer for 10 min and subjected to SDS-PAGE gels. After wet-transferred with 200 mA for 80 min, the nitrocellulose membrane was incubated with 1 mL condensed preliminary antibodies in 10 mL TBST buffer at 4 °C overnight. After washed with TBST and ddH_2_O, the membrane was incubated with 100 mM glycine (pH = 2.7 and 1.9, 9 mL each) for antibody elution, and neutralized with 1 M Tris-HCl (pH = 8.8). For detecting endogenous VISP1, 0.1 g *Arabidopsis* leaves were homogenized in 200 μL extraction buffer [100 mM Tris-HCl (pH 7.5), 400 mM sucrose, 1 mM EDTA (pH 8.0), 1 mM PMSF, 4% (v/v) cocktail]. The mixture was incubated on ice for 40 min and centrifuged at 13,523 *g* for 10 min at 4 °C. The supernatant was subjected to Tris-Tricine-SDS-PAGE gels according to the protocol (Solarbio, Beijing Chia). Immunoblotting analyses were performed using the purified anti-VISP1 antibodies (1:200).

### RNA extraction, DNA isolation, and RT-qPCR analysis

Total RNA was isolated from 0.1 g mock- or virus-infected plant leaves using Trizol reagent (Invitrogen, Carlsbad, CA, USA) according to the manufacturer’s instructions. For RT-qPCR assays, 2.5 μg total RNA was firstly treated with *DNase* I for 40 min at 37 °C, and then the first strand cDNA was synthesized from the treated RNA by using Oligo(dT) 20 primer and M-MLV reverse transcriptase (Promega, USA) following the recommended protocol. QPCR assays were carried out with the 2×SsoFast EvaGreen Supermix (Bio-Rad) and specific primers listed in Supplementary Data 1. The Arabidopsis *Actin2* gene served as an endogenous control. Total DNA was extracted from infected *Arabidopsis* plants using the CTAB method. Extracted DNA (800 ng) were determined by qPCR using specific primers and normalized to *Actin2* as an internal genomic DNA control.

### Quantification and statistical analysis

All experiments were repeated at least three times, and representative results are shown. Intensities of protein bands detected by immunoblotting analysis were quantified using ImageJ software. For all assays, means and standard deviation (SD) values were calculated, and significances were determined by Student’s *t* test or ANOVA followed by Tukey’s or Dunnett’s multiple comparison test.

### Accession numbers

Gene sequences from this study can be found in GenBank/EMBL libraries under the following accession numbers: *AtVISP1*, MT063056; *AtSGS3*, NM_122263; *AtATG5*, NM_121735.5; *AtATG7*, NM_123958.3; *Actin2*, AY096381; *CMV-RNA1*, NC_002034; *CMV-RNA2*, NC_002035; *CMV-RNA3*, NC_001440; *BSCTV*, KX867036; *C2*, CAA65840; *CaLCuV-A*, U65529; *CaLCuV-B*, MH359397; *AC2*, NP_620887; *TBSV P19*, AJ288926; *PoLV P14*, AB602348.

### Reporting summary

Further information on research design is available in the Nature Portfolio Reporting Summary linked to this article.

## Supplementary information

Supplementary Information

Description of Additional Supplementary Files

Supplementary Data 1.

Reporting Summary

## Data Availability

The source data for Figs. 1−6, and Supplementary Figs. 1, 3−11 are provided as a Source Data file. All other data that support the findings of this study are available from the corresponding author upon request. Source data are provided with this paper.

## Source data

Source Data
