# Supplementary material for: A selective autophagy receptor VISP1 induces symptom recovery by targeting viral silencing suppressors
Source: Nat Commun. 2023 Jun 29;14:3852. doi: 10.1038/s41467-023-39426-0 (PMC10310818; doi:10.1038/s41467-023-39426-0)
Supplement: Supplementary file 1 — Supplementary Information [file 41467_2023_39426_MOESM1_ESM.pdf]

Supplementary materials for

A selective autophagy receptor VISP1 induces symptom recovery by targeting viral silencing suppressors

Xin Tong[1, 2]†, Jia-Jia Zhao[1]†, Ya-Lan Feng[1], Jing-Ze Zou[1], Jian Ye[3], Junfeng Liu[2], Chenggui Han[2], Dawei Li[1], Xian-Bing Wang[1]*

[1] State Key Laboratory of Plant Environmental Resilience, College of Biological Sciences, China Agricultural University, Beijing 100193, China

[2] College of Plant Protection, China Agricultural University, Beijing 100193, China

[3] State Key laboratory of Plant Genomics, Institute of Microbiology, Chinese Academy of Sciences, Beijing, China

† These authors contributed equally to this work.

*Corresponding author. Email: wangxianbing@cau.edu.cn

Supplementary Information

Supplementary Figure. 1-12

Supplementary Fig. 1

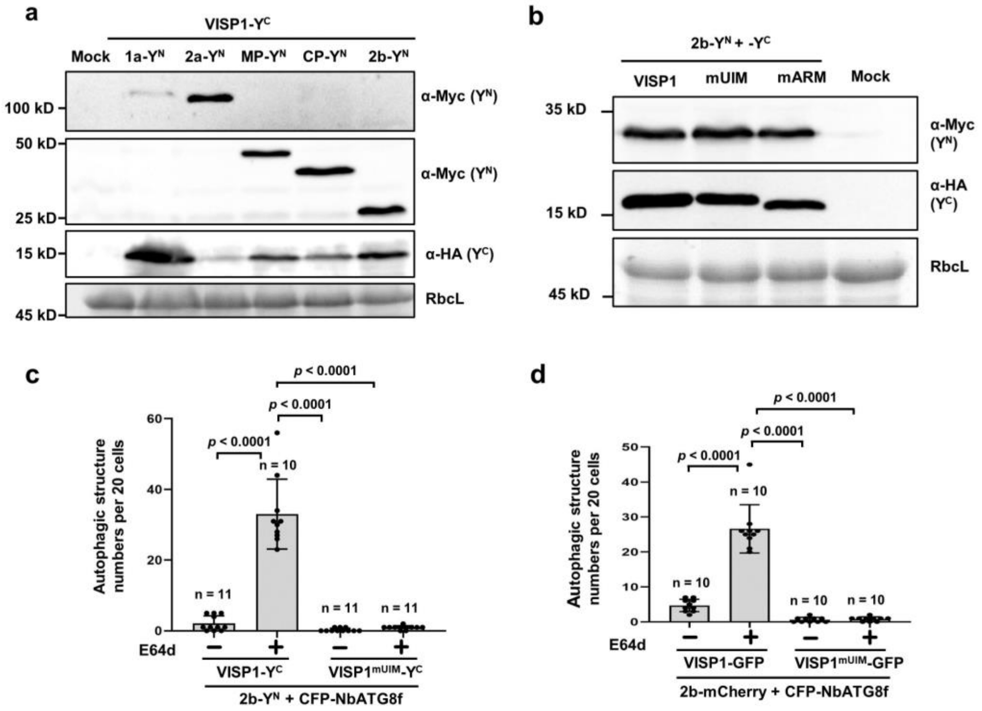

Supplementary Fig. 1. Immunoblotting analyses detecting protein accumulation of BiFC assays. a and **b,** *N. benthamiana* leaves were collected at 60 hpi with agroinfiltration for immunoblotting analyses. The Y^N- and Y^C-tagged proteins were detected with anti-Myc and anti-HA antibodies, respectively. **c** and **d**, Numbers of autophagic structures per 20 cells in **Fig. 1c** and **1d**. n = 10 or 11 images per treatment. The data represent the mean ± SD. The *p* values by one-way ANOVA multiple comparisons followed by Tukey's multiple comparisons test are indicated in the source data, $p < 0.05$.

Supplementary Fig. 2

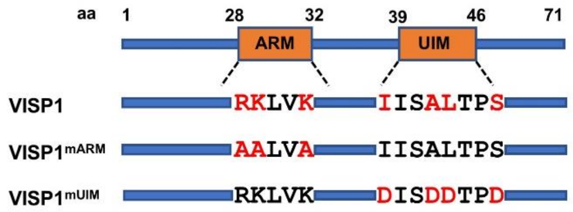

Supplementary Fig. 2. Schematic representation of Arabidopsis VISP1 and substitution mutants in the UIM and ARM motifs. UIM, ubiquitin-interacting motif. ARM, arginine/lysine-rich motif. Note that VISP1^{mUIM} does not interact with ATG8s, and VISP1^{mARM} does not interact with substrates.

Supplementary Fig. 3

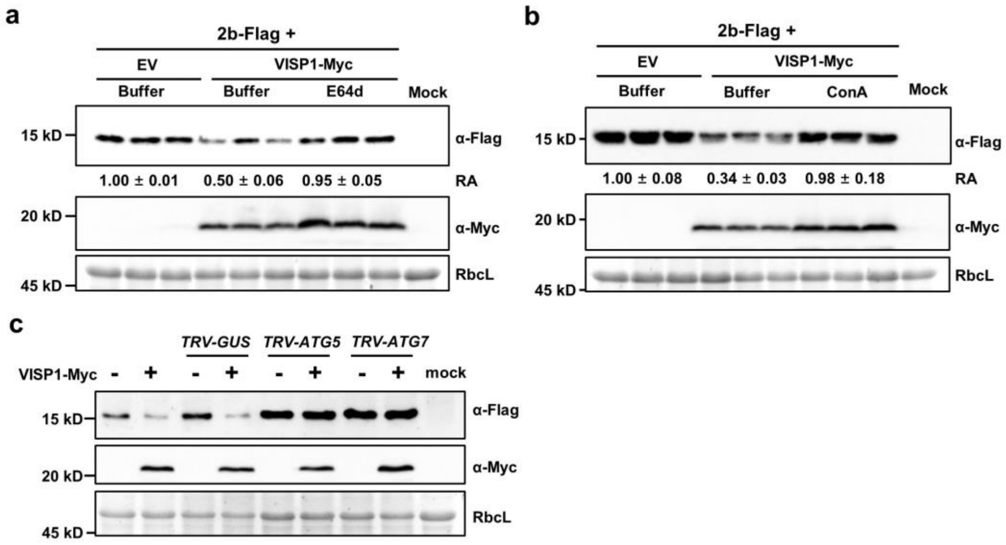

Supplementary Fig. 3. VISP1 mediates autophagic degradation of 2b. a and **b,** Effects of VISP1-Myc and autophagy inhibitors on accumulation of 2b-Flag in *N. benthamiana* leaves. The co-infiltrated leaves were treated with buffer or 100 µM E64d (**a**) or 1 µM ConA (**b**) at 48 hpi and harvested for immunoblotting analyses at 60 hpi. **c** Immunoblotting analyses detecting accumulation of 2b-Flag co-expressed with empty vector or VISP1-Myc in *N. benthamiana* leaves with knocking down of *NbATG5* and *NbATG7* induced by TRV-VIGS. TRV-GUS served as a negative control.

Supplementary Fig. 4

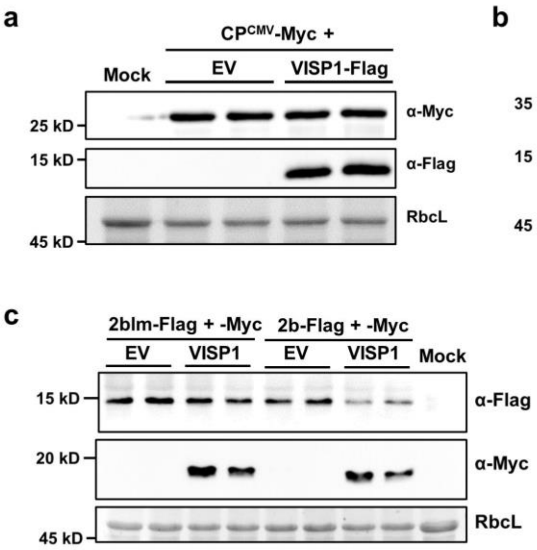

Supplementary Fig. 4. Effects of VISP1 on CP, MP, 2blm, and 2b protein accumulation in *N. benthamiana* leaves. a,b Immunoblotting analyses detecting accumulation of CP^{CMV}-Myc or MP^{CMV}-Myc co-expressed with VISP1-Flag in *N. benthamiana* leaves. **c** Immunoblotting analyses detecting accumulation of 2blm-Flag or 2b-Flag co-expressed with VISP1-Myc in *N. benthamiana* leaves. RbcL served as loading controls.

Supplementary Fig. 5

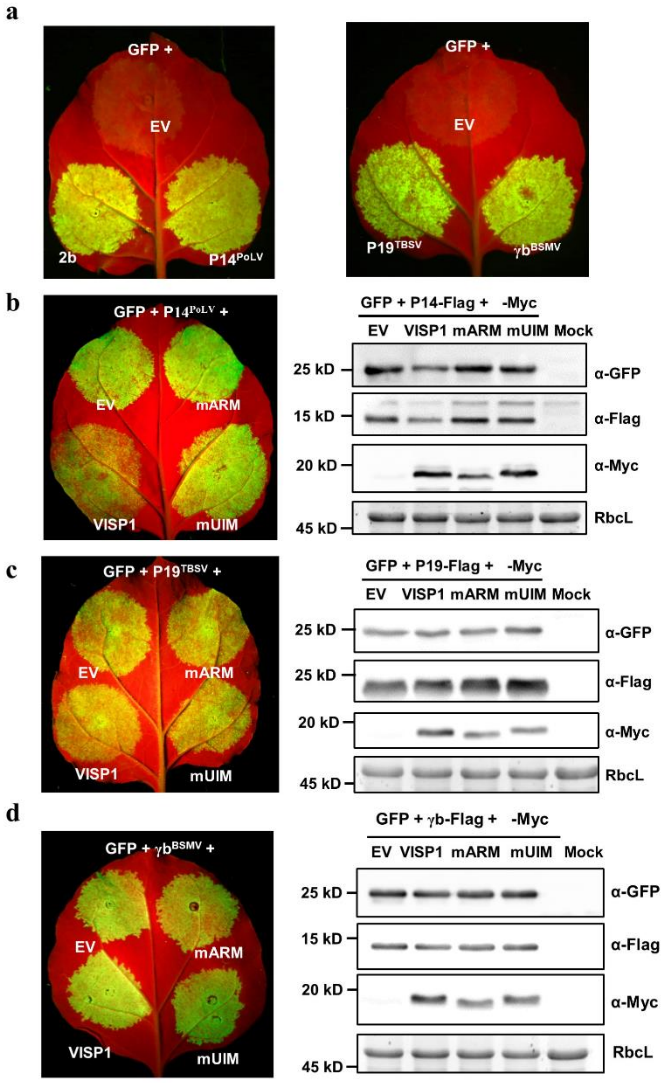

Supplementary Fig. 5. Effects of VISP1 on silencing suppression and protein accumulation of several VSRs. a-d, GFP fluorescence in regions of *N. benthamiana* leaves co-expressing GFP, with empty vector (EV), CMV 2b, PoLV P14, TBSV P19, or BSMV γb. Effects of VISP1 on silencing suppression and protein accumulation of PoLV P14, TBSV P19, and BSMV γb. The Flag-tagged VSRs and GFP were co-expressed with VISP1-Myc/VISP1^{mARM}-Myc/VISP1^{mUIM}-Myc/EV. The infiltrated leaves were photographed with a long-wave UV light at 5 dpi.

Supplementary Fig. 6

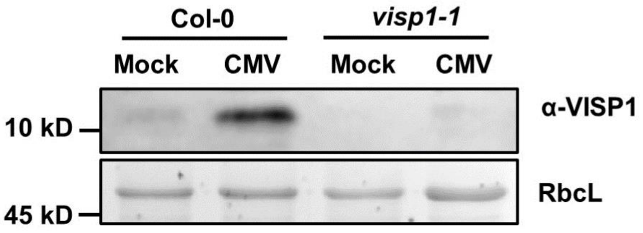

Supplementary Fig. 6. Immunoblotting analyzing expression of endogenous VISP1. The systemically infected leaves of Col-0 and *visp1-1* inoculated with CMV were collected at 7 dpi, and were detected accumulation of endogenous VISP1 protein by immunoblotting analyses with purified anti-VISP1 antibodies.

Supplementary Fig. 7

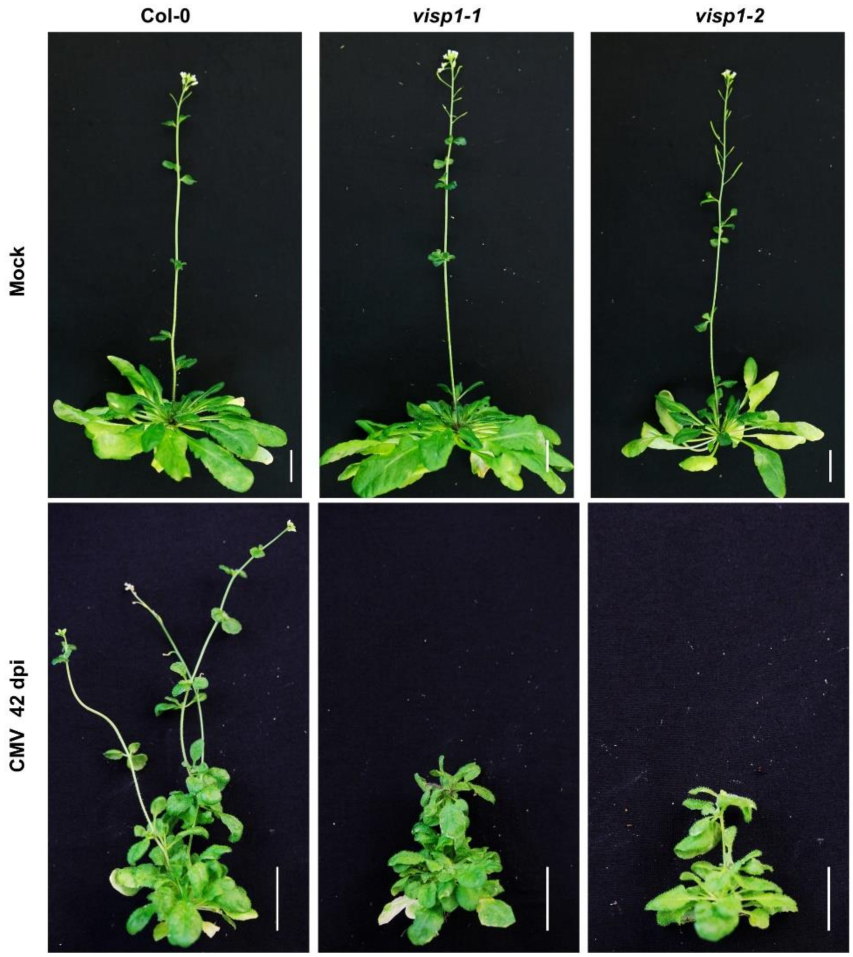

Supplementary Fig. 7. Developmental phenotype and pathogenic response of Col-0 and *visp1* mutants. Col-0, *visp1-1*, and *visp1-2* plants were inoculated with mock buffer and wild-type CMV and photographed at 42 dpi. Scale bars, 2 cm.

Supplementary Fig. 8

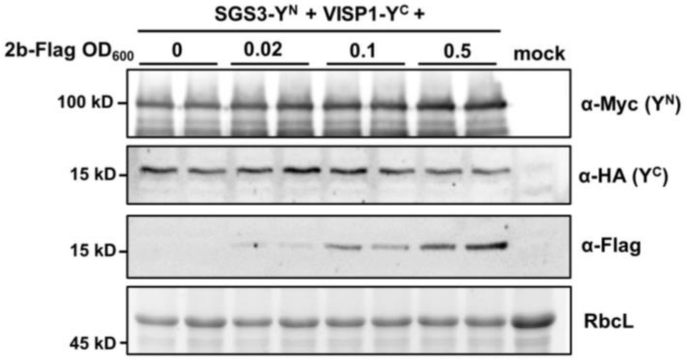

Supplementary Fig. 8. Immunoblotting analyses detecting protein accumulation in the competitive BiFC assays of Figure 5c. SGS3-Y^N and VISP1-Y^C were detected by anti-Myc and -HA antibodies, respectively. 2b-Flag was detected by anti-Flag antibodies.

Supplementary Fig. 9

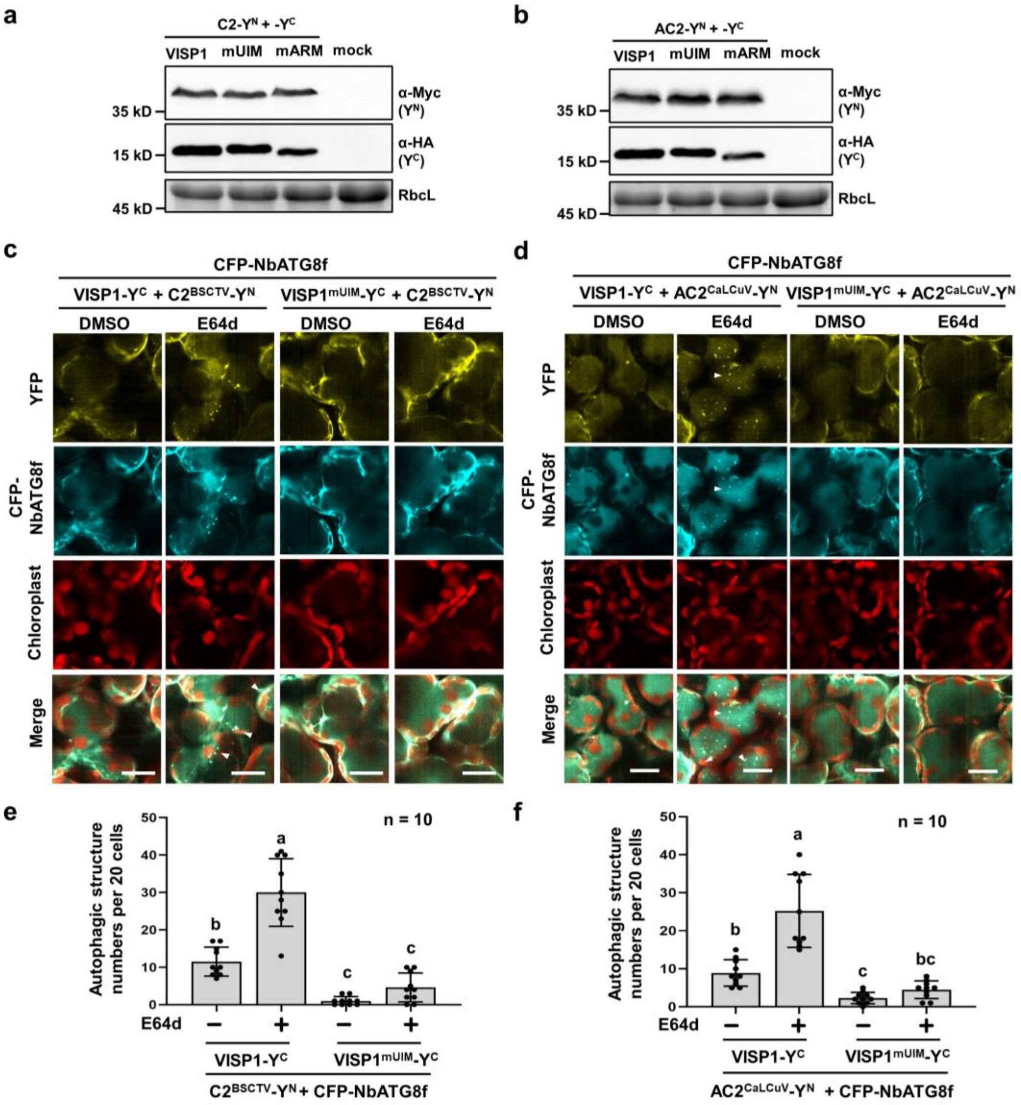

Supplementary Fig. 9. VISP1-C2/AC2 interacting bodies were colocalized with CFP-NbATG8f. a and b, immunoblotting analyses for detecting protein accumulation in the BiFC assays of Figure 6**a.** The Y^N- and Y^C-tagged proteins were detected with anti-Myc and anti-HA antibodies, respectively. **c** and **d**, Confocal analysis of the C2^{BSCTV}-Y^N/VISP1-Y^C, and AC2^{CaLCuV}-Y^N/VISP1-Y^C-formed bodies co-localized with CFP-NbATG8f-labelled autophagic bodies in *N. benthamiana* leaves. VISP1^{mUIM}-Y^C served as a negative control. The infiltrated leaves were treated with 100 µM E64d or DMSO at 48 hpi and photographed at 60 hpi. Scale bars, 20 µm. **e and f**, Numbers of autophagic structures per 20 cells in **c** and **d**. Values represent means from 10 images per treatment. The data represent the mean ± SD and *p* values by one-way ANOVA multiple comparisons followed Dunnett's multiple comparisons test are indicated in the source data.

Supplementary Fig. 10

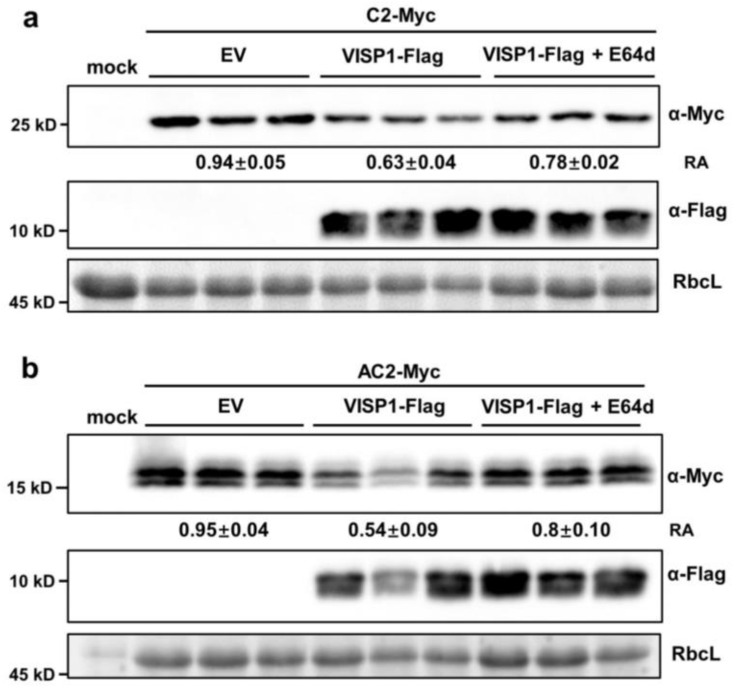

Supplementary Fig. 10. VISP1 mediates autophagic degradation of the C2/AC2 suppressors. **a** and **b**, effects of VISP1-Flag and inhibitor E64d on accumulation of C2-Myc or AC2-Myc in *N. benthamiana* leaves. The co-infiltrated leaves were treated with or without 100 µM E64d at 48 hpi and were harvested for immunoblotting analyses at 60 hpi.

Supplementary Fig. 11

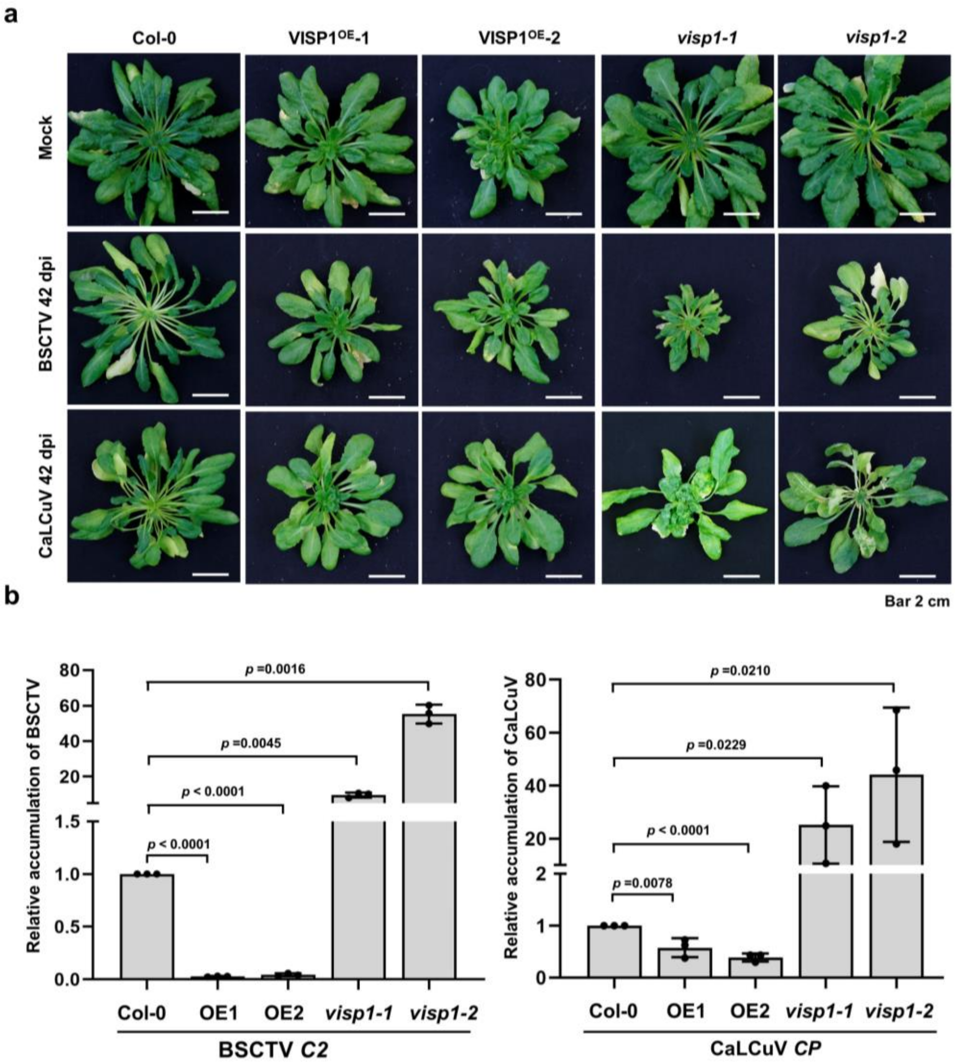

Supplementary Fig. 11. VISP1 inhibits late infections of BSCTV and CaLCuV. a Developmental phenotypes and pathogenic responses of Col-0, VISP1^{OE} and *visp1* mutant plants inoculated with mock buffer, BSCTV, or CaLCuV at 42 dpi. Scale bars, 2 cm. **b** Relative virus accumulation of BSCTV or CaLCuV in Col-0, VISP1^{OE}, and *visp1* plants at 42 dpi. The specific primers corresponding to BSCTV C2 or CaLCuV CP were used for qPCR. *Actin2* as an internal genomic DNA control. Viral accumulation in Col-0 plants were set as one unit. Error bars indicate SD (n = 3). *p* values by one-tailed Student's *t* test are indicated.

Supplementary Fig. 12

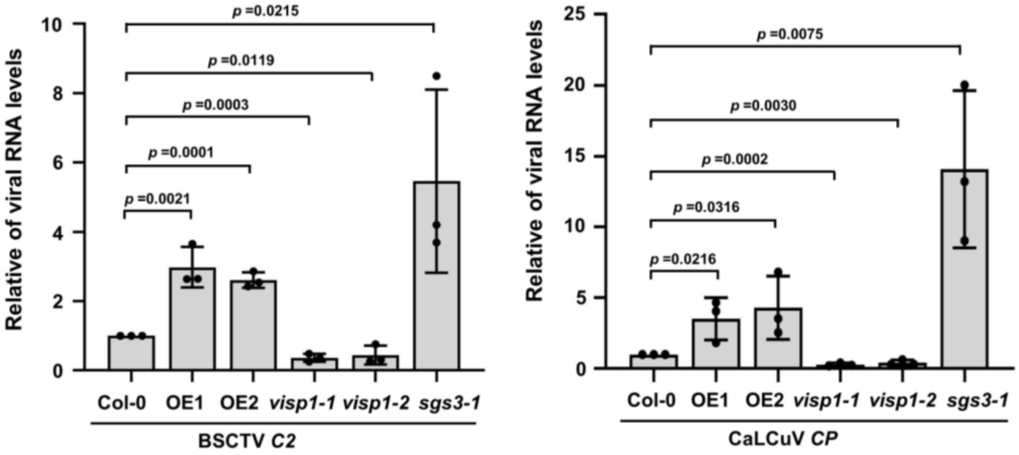

Supplementary Fig. 12. VISP1 facilitates early infections of BSCTV and CaLCuV in inoculated leaves. RT-qPCR analyzing relative viral mRNA of BSCTV and CaLCuV in inoculated leaves of Col-0, VISP1^{OE}, *visp1*, and *sgs3-1* plants at 3 dpi. The specific primers corresponding to BSCTV C2 or CaLCuV CP were used for qPCR. The *Actin2* mRNA served as an internal control. Viral accumulation in Col-0 plants were set as one unit. Error bars indicate SD (n = 3). *p* values by one-tailed Student's *t* test are indicated.
