# Supplementary material for: A selective autophagy receptor VISP1 induces symptom recovery by targeting viral silencing suppressors
Source: Nat Commun. 2023 Jun 29;14:3852. doi: 10.1038/s41467-023-39426-0 (PMC10310818; doi:10.1038/s41467-023-39426-0)
Supplement: Supplementary file 2 — Description of Additional Supplementary Files [file 41467_2023_39426_MOESM2_ESM.pdf]

Description of Additional Supplementary Files:

Supplementary Data 1. Primers used in this study
