# Supplementary material for: A selective autophagy receptor VISP1 induces symptom recovery by targeting viral silencing suppressors
Source: Nat Commun. 2023 Jun 29;14:3852. doi: 10.1038/s41467-023-39426-0 (PMC10310818; doi:10.1038/s41467-023-39426-0)
Supplement: Supplementary file 4 — Reporting Summary [file 41467_2023_39426_MOESM4_ESM.pdf]

nature portfolio

Corresponding author(s): Xian-Bing Wang

Last updated by author(s): 2023-6-5

Reporting Summary

Nature Portfolio wishes to improve the reproducibility of the work that we publish. This form provides structure for consistency and transparency in reporting. For further information on Nature Portfolio policies, see our Editorial Policies and the Editorial Policy Checklist.

Statistics

For all statistical analyses, confirm that the following items are present in the figure legend, table legend, main text, or Methods section.

n/a	Confirmed	
☐	☒	The exact sample size (*n*) for each experimental group/condition, given as a discrete number and unit of measurement
☐	☒	A statement on whether measurements were taken from distinct samples or whether the same sample was measured repeatedly
☐	☒	The statistical test(s) used AND whether they are one- or two-sided<br>*Only common tests should be described solely by name; describe more complex techniques in the Methods section.*
☐	☒	A description of all covariates tested
☐	☒	A description of any assumptions or corrections, such as tests of normality and adjustment for multiple comparisons
☐	☒	A full description of the statistical parameters including central tendency (e.g. means) or other basic estimates (e.g. regression coefficient) AND variation (e.g. standard deviation) or associated estimates of uncertainty (e.g. confidence intervals)
☐	☒	For null hypothesis testing, the test statistic (e.g. F, t, r) with confidence intervals, effect sizes, degrees of freedom and P value noted<br>*Give P values as exact values whenever suitable.*
☒	☐	For Bayesian analysis, information on the choice of priors and Markov chain Monte Carlo settings
☐	☒	For hierarchical and complex designs, identification of the appropriate level for tests and full reporting of outcomes
☒	☐	Estimates of effect sizes (e.g. Cohen's *d*, Pearson's *r*), indicating how they were calculated

Our web collection on statistics for biologists contains articles on many of the points above.

Software and code

Policy information about availability of computer code

Data collection	We used TAIR10 (http://arabidopsis.org) and NCBI (https://www.ncbi.nlm.nih.gov/nuccore/) to obtain the information of all the genes.<br>Zeiss LSM 880 confocal microscope for confocal observation.<br>Azure Biosystems C600 was used for obtain bands of immunoblotting analyses.<br>qPCR experiments were measured and analyzed with bio-rad CFX_Manager 3.0.
Data analysis	We used Prism8 (GraphPad) to dispose the statistics, and ImagJ to qualify the bands. Bio-rad CFX_Manager 3.0 for analyzing RT-qPCR results.<br>ZEN 2012 was used for microscopy. Quantity One 4.6.2 for obtaining loading control RbcL in the immunoblotting analyses.

For manuscripts utilizing custom algorithms or software that are central to the research but not yet described in published literature, software must be made available to editors and reviewers. We strongly encourage code deposition in a community repository (e.g. GitHub). See the Nature Portfolio guidelines for submitting code & software for further information.

Data

Policy information about availability of data

All manuscripts must include a data availability statement. This statement should provide the following information, where applicable:

- Accession codes, unique identifiers, or web links for publicly available datasets
- A description of any restrictions on data availability
- For clinical datasets or third party data, please ensure that the statement adheres to our policy

We have provided a full data availability statement in the manuscript.

Human research participants

Policy information about studies involving human research participants and Sex and Gender in Research.

Reporting on sex and gender	Not applicable
Population characteristics	Not applicable
Recruitment	Not applicable
Ethics oversight	Not applicable

Note that full information on the approval of the study protocol must also be provided in the manuscript.

Field-specific reporting

Please select the one below that is the best fit for your research. If you are not sure, read the appropriate sections before making your selection.

☒ Life sciences ☐ Behavioural & social sciences ☐ Ecological, evolutionary & environmental sciences

For a reference copy of the document with all sections, see nature.com/documents/nr-reporting-summary-flat.pdf

Life sciences study design

All studies must disclose on these points even when the disclosure is negative.

Sample size	The bars on the pictures of plants cells are produced by the imaging software automatically. The size of plants are indicated by the reference photographed with plants at the same time.
Data exclusions	No data were excluded from our analyses.
Replication	All main experiments were repeated three times.
Randomization	For the detection of virus accumulation, systematically infected leaves were collected randomly among groups.
Blinding	Analysis were performed in a manner blinded to treatments in all experiments.

Reporting for specific materials, systems and methods

We require information from authors about some types of materials, experimental systems and methods used in many studies. Here, indicate whether each material, system or method listed is relevant to your study. If you are not sure if a list item applies to your research, read the appropriate section before selecting a response.

Materials & experimental systems

n/a	Involved in the study
☐	☒ Antibodies
☒	☐ Eukaryotic cell lines
☒	☐ Palaeontology and archaeology
☒	☐ Animals and other organisms
☒	☐ Clinical data
☒	☐ Dual use research of concern

Methods

n/a	Involved in the study
☒	☐ ChIP-seq
☒	☐ Flow cytometry
☒	☐ MRI-based neuroimaging

Antibodies

Antibodies used	Rabbit polyclonal anti-FLAG, Sigma Cat. No. F7425. Mouse monoclonal anti-His, Proteintech, Cat#66005-1-lg. Mouse monoclonal anti-HA, MEDICAL&BIOLOGICAL LABORATORIES CO.,LTD, Cat#M180-3. Rabbit polyclonal anti-GST, Abcam, Cat#ab9085. Rabbit polyclonal anti-myc, EASYBIO, Cat#BE2011. Goat anti-Mouse IgG (H+L)-HLP Conjugate, BIO-RAD, Cat#170-6516. Goat anti-Rabbit IgG (H+L)-HLP Conjugate, EASYBIO, Cat#BE0101.
Validation	The validation of antibodies were proved in this manuscript and our previous paper (Zhang et al.,Elife,2020, 9, e53753).
